# Emerging Targets and Therapeutics in Immuno-Oncology: Insights from Landscape Analysis

**DOI:** 10.1021/acs.jmedchem.4c00568

**Published:** 2024-05-24

**Authors:** Kavita A. Iyer, Julian Ivanov, Rumiana Tenchov, Krittika Ralhan, Yacidzohara Rodriguez, Janet M. Sasso, Sabina Scott, Qiongqiong Angela Zhou

**Affiliations:** † ACS International India Pvt. Ltd., Pune 411044, India; ‡ CAS, A Division of the American Chemical Society, Columbus, Ohio 43210, United States

## Abstract

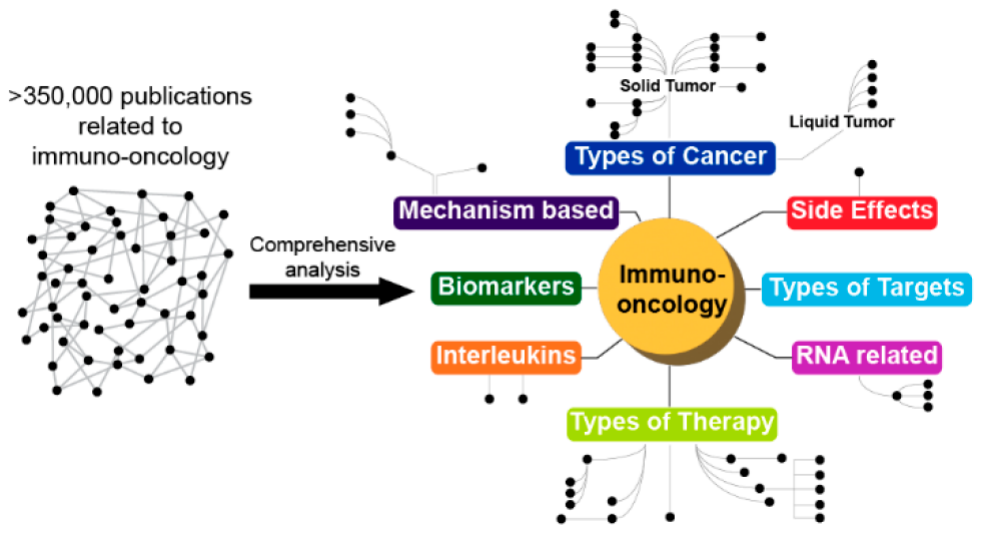

In the ever-evolving landscape of cancer research, immuno-oncology stands as a beacon of hope, offering novel avenues for treatment. This study capitalizes on the vast repository of immuno-oncology-related scientific documents within the CAS Content Collection, totaling over 350,000, encompassing journals and patents. Through a pioneering approach melding natural language processing with the CAS indexing system, we unveil over 300 emerging concepts, depicted in a comprehensive “Trend Landscape Map”. These concepts, spanning therapeutic targets, biomarkers, and types of cancers among others, are hierarchically organized into eight major categories. Delving deeper, our analysis furnishes detailed quantitative metrics showcasing growth trends over the past three years. Our findings not only provide valuable insights for guiding future research endeavors but also underscore the merit of tapping the vast and unparalleled breadth of existing scientific information to derive profound insights.

## Significance

A “Trend Landscape Map” of emerging concepts in immuno-oncology has been crafted based on a comprehensive analysis of the extensive CAS Content Collection. The map has been constructed by utilizing a novel Natural Language Processing algorithm in combination with extensive curation by subject matter experts, resulting in identification of >300 emergent topics across eight main areas of interest. The map acts as a visual aid, with detailed quantitative metrics of recent growth illustrating the spread of emerging ideas in immuno-oncology.

## Introduction

**Figure 1:**
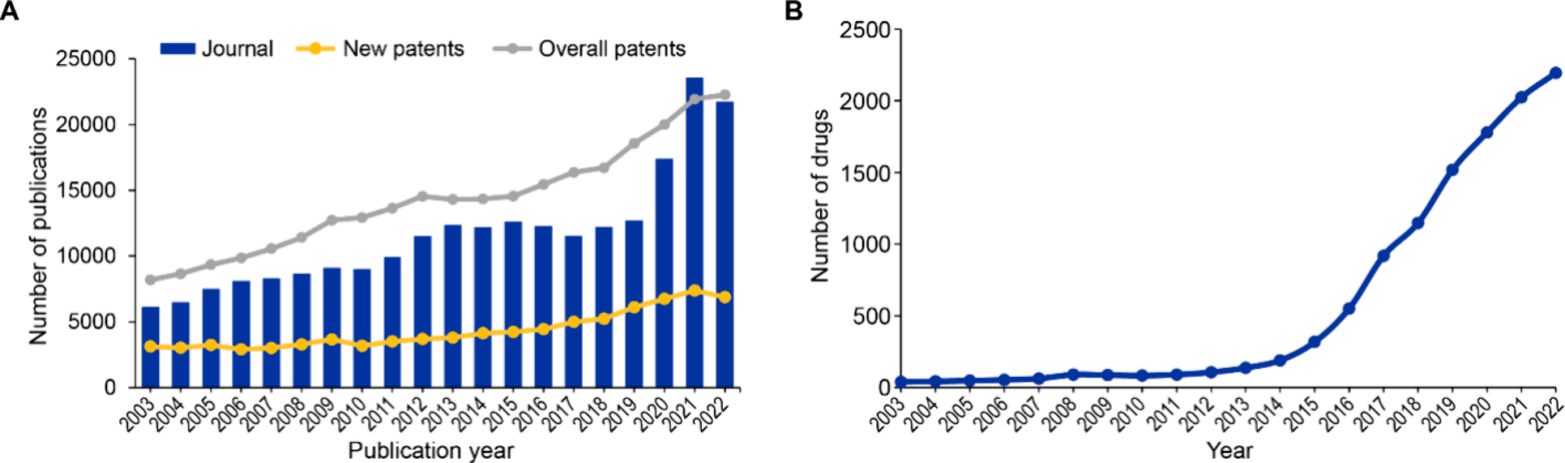
(A) Overall growth in publications (journals and patents) pertaining to immuno-oncology from the CAS Content Collection over the past two decades (2003–2022). The blue bars represent journal publications while the yellow line represents new patent publications, and the gray line represents the total number of patent publications reflective of overall patent activity. (B) Growth in the number of drugs in the field of immuno-oncology in the preclinical stage over the past two decades (2003–2022); data retrieved from Pharmaprojects.

Cancer has been declared as one of the leading causes of death by the World Health Organization (WHO).^1^ The global economic burden of cancer is undeniable, with projections estimating nearly $25 trillion in the year 2050.^2^ In the United States alone, ∼600K cancer deaths were estimated for 2023.^3^ In this bleak landscape, immuno-oncology—a field at the forefront of cancer research and treatment—has emerged as a beacon of hope in the fight against various types of cancers. Harnessing the body’s immune system to recognize, target, and eliminate tumor cells, immuno-oncology’s extraordinary promise and rapid growth have captured the attention of researchers and pharmaceutical industries worldwide. This burgeoning potential and heightened interest are vividly reflected in the growing number of scientific publications (Figure 1A), especially in the past four years, and the escalating number of drugs under evaluation in clinical trials since 2015 (Figure 1B), accounting for ∼5,500 clinical trials currently ongoing across various clinical phases.

Among the better-known cancer immunotherapies are immune checkpoint inhibitors (ICIs) and antibody–drug conjugates (ADCs). The discovery and development of ICIs has been revolutionary for cancer therapy and has led to a paradigm shift.^4^ The therapeutic success of U.S. Food and Drug Administration (FDA)-approved ICIs Keytruda^5^ (pembrolizumab; Merck & Co.) and Opdivo^6^ (nivolumab; Bristol-Myers Squibb) in treating melanoma^7^ and non-small-cell lung cancer (NSCLC)^8^ among other types of cancer has been encouraging. Besides ICIs, U.S. FDA-approved ADCs such as Kadcyla^9^ (ado-trastuzumab emtansine; Genentech Inc.), Besponsa^10^ (inotuzumab ozogamicin; Pfizer Inc.), and Elahere^11^ (mirvetuximab soravtansine; ImmunoGen Inc.) have also shown tremendous success in the treatment of metastatic breast cancer,^12^ acute lymphoblastic leukemia (ALL),^13^ and ovarian cancer,^14^ respectively. The remarkable success of ADCs^15^ has meant that the scope of ADCs is expanding beyond the boundaries of cancer.

Out of the 55 novel drugs that gained U.S. FDA approval in 2023 alone,^16^ over 12% are associated with cancer immunotherapy, including the monoclonal antibodies (Loqtorz/toripalimab-tpzi^17^ and Zynyz/retifanlimab-dlwr^18^) against the immune checkpoint molecule programmed cell death 1 receptor (PD-1) and bispecific T-cell engagers directed toward CD20 (Columvi/glofitamab^19^) and B-cell maturation antigen (BCMA) (Elrexfio/elranatamab-bcmm^20^). The therapeutic and commercial success of ICIs and ADCs and the continued regulatory approvals of new cancer immunotherapeutic drugs have translated to an impetus for pharmaceutical companies to continue investing in this field with a sustained interest in developing newer immunotherapeutic drugs. This is exemplified by the nearly $16 billion in investments for cancer immunotherapy in 2022–2023 (Pitchbook^21^).

The pursuit of advancements in immuno-oncology by academic and commercial organizations has led to proliferative, sustained, and rampant expansion of journal and patent publications. In this report, leveraging the extensive CAS Content Collection, we delved deep into this fast-growing corpus of scientific publications, aiming to identify emerging trends that will be invaluable to researchers in this vibrant community. We utilized a novel natural language processing (NLP)-based algorithm^22^ in combination with extensive curation by subject matter experts, resulting in quantitative identification of >300 emergent topics across eight main areas of interest. Moreover, our analysis allowed us to capture ideas that appear to be in the very early/nascent stages of emergence. We have designed a trend landscape map to illustrate the spread of emerging ideas in immuno-oncology across a wide range of topics with an emphasis on their growth over the past 3 years.

## Trend Landscape Map: A Bird’s Eye View of Emerging Concepts in Immuno-Oncology

Our data set comprises ∼350K publications extracted from the CAS Content Collection, the largest human-compiled multidisciplinary database of published documents and substances, employing a comprehensive search query developed by subject matter experts. The extracted data, consisting of a wide range of information, including extensive bibliographic information, CAS indexed concepts, and substances, was subjected to NLP^22^ to identify frequently used phrases (for a detailed description of the methods, see the Supporting Information and Figure S1). Subsequently, these phrases underwent exhaustive manual scrutiny by subject matter experts, forming the basis for calculating the relative rates of publication growth over the period 2020–2022.

**Figure 2:**
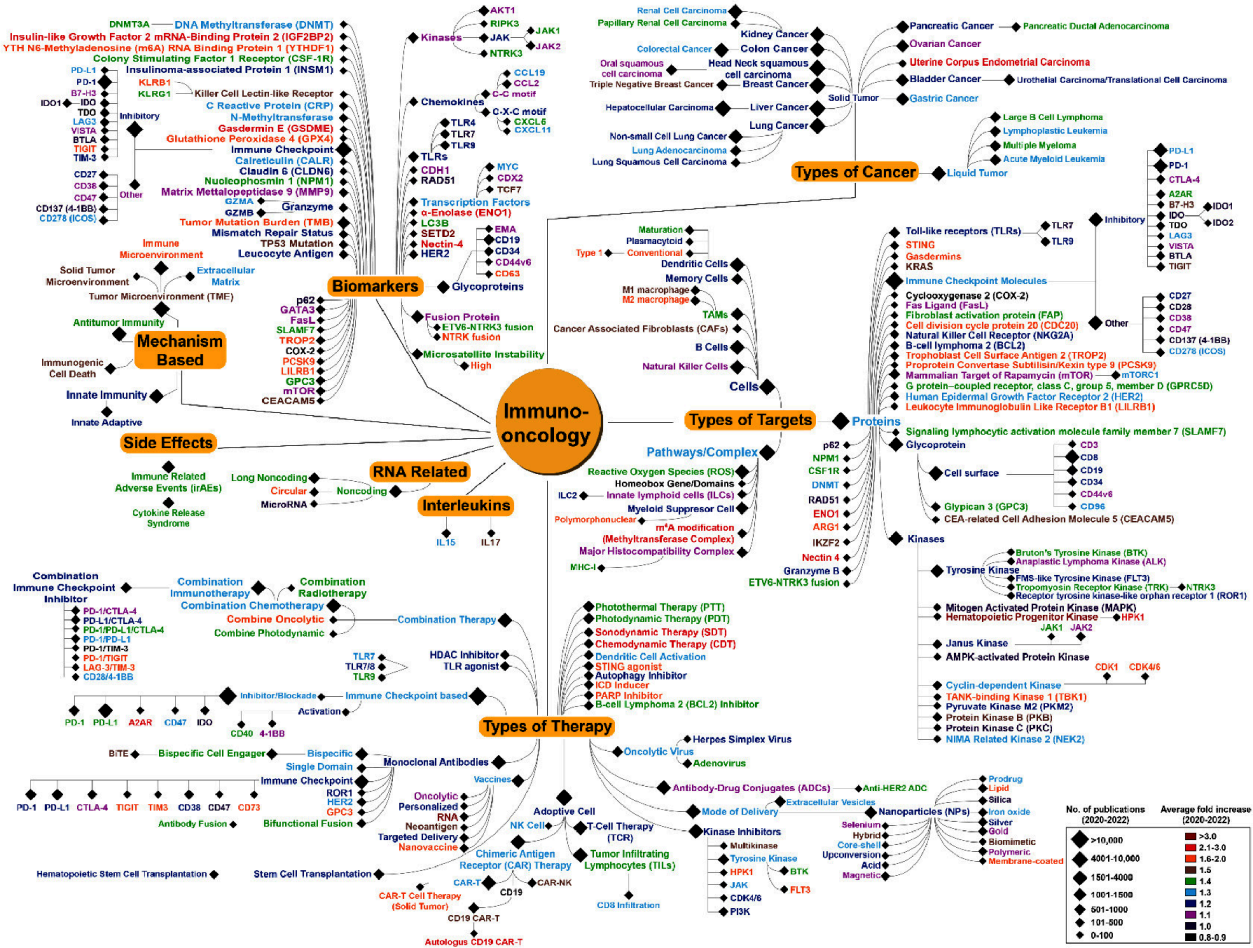
Detailed “Trend Landscape Map” of emerging concepts/ideas associated with immuno-oncology curated from NLP data analysis on >350,000 publications from the CAS Content Collection. The size of the symbol (◆) corresponds to the number of publications for the period 2020–2022, while the color of the text indicates the average fold increase in publications for the period 2020–2022, an indicator of growth. For a detailed description of average fold increase, please see Methods in the Supporting Information.

**Figure 3:**
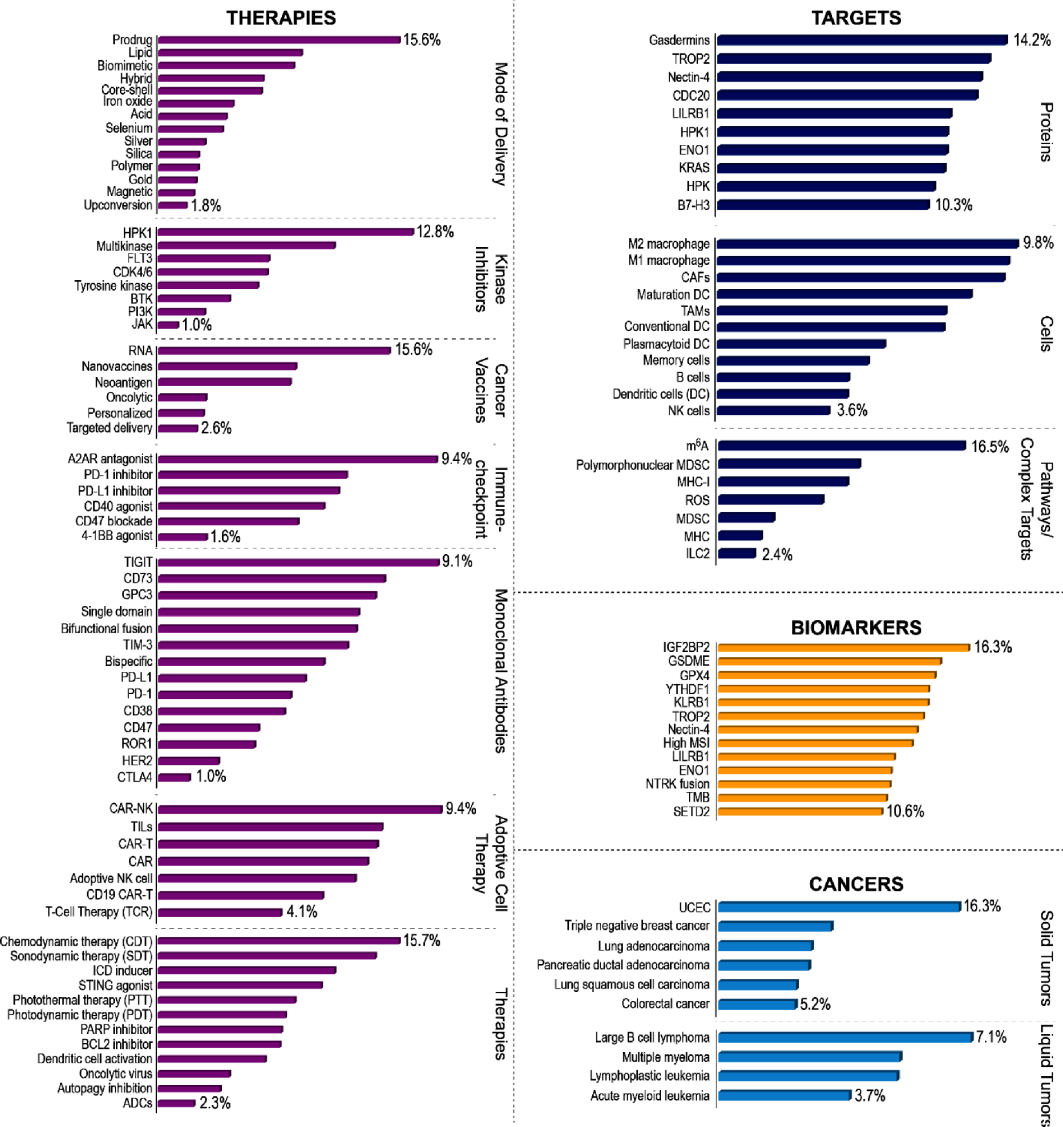
Fastest growing concepts in a few selected major immuno-oncology-related categories from NLP data analysis on >350,000 publications from the CAS Content Collection. Data includes both journal and patent publications, expressed as relative growth rates (which are average publication rates normalized with respect to number of documents) for the period 2020–2022. For more detail, please see Methods in the Supporting Information.

The identified emerging concepts were categorized into eight major domains: targets, therapies, interleukins, RNA-related, side effects, mechanism-based, biomarkers, and types of cancer. To visually represent these trending and emerging concepts, we constructed a trend landscape map (Figure 2) featuring a hierarchical arrangement of emerging concepts within pertinent groups and incorporated data from journal and patent publications. Most identified concepts ended up being clustered in the following four categories: types of targets, therapies, cancers, and biomarkers. The trend landscape map has been designed to provide, at a glance, the average fold increase in publications over 2020–2022 and the number of publications over 2020–2022 using a color scale and symbols, respectively. Broadly speaking, the identified concepts grew at a modest (1.1 to 1.5×), fast (1.6 to 2×), or very fast (2 to >3×) pace. In addition, for the reader’s ease, we have highlighted the relative growth rates of the fastest growing concepts in each of the four most prolific categories: types of targets, biomarkers, cancers, and therapies (see Figure 3). Both average fold increase and relative growth rate are metrics indicating scientific interest albeit expressed differently, with the latter being normalized with respect to number of publications.

**Figure 4:**
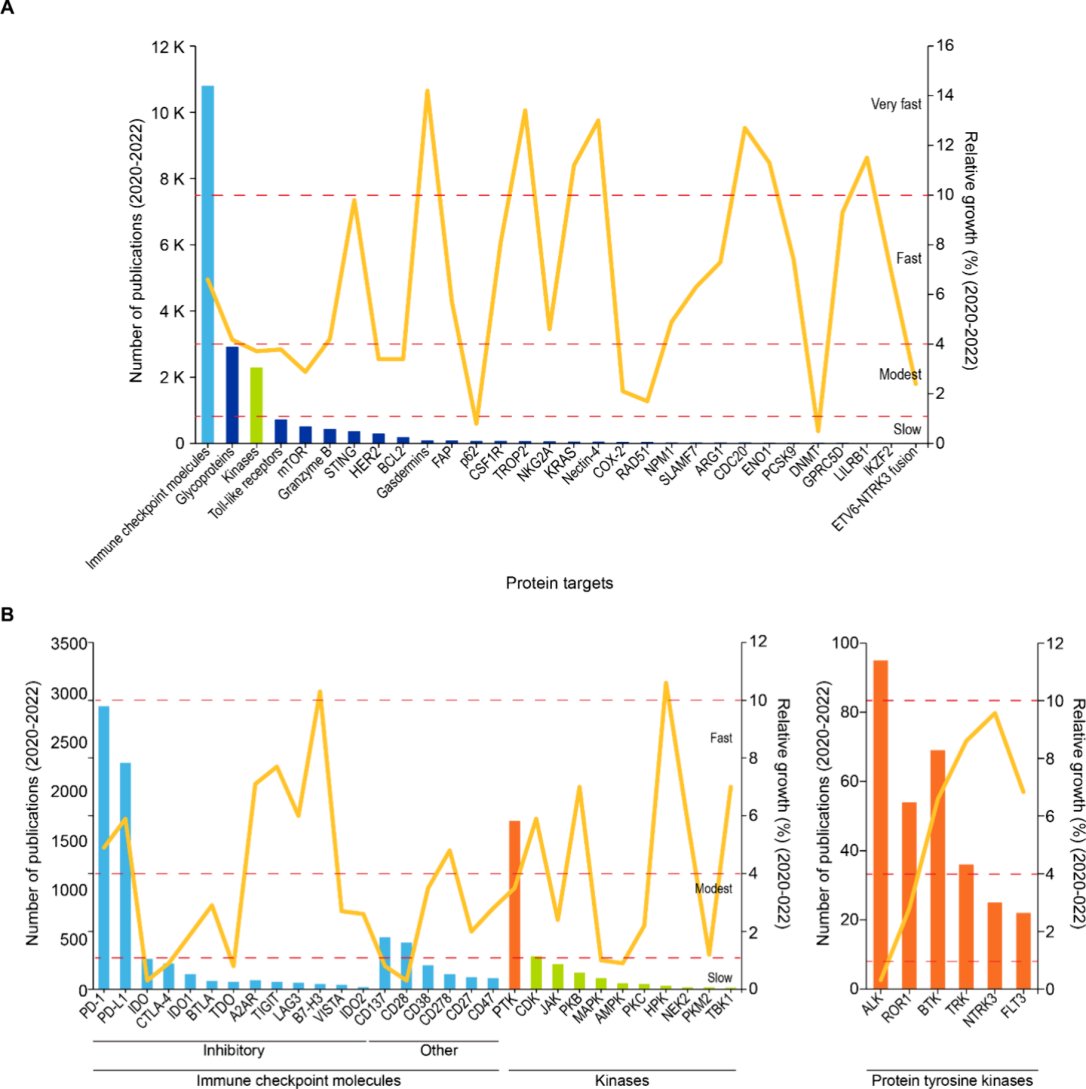
Comparison between relative growth rates (shown as yellow line) and number of publications (shown as colored bars) of (A) protein targets and (B) individual immune checkpoint molecules and kinase protein targets. Blue and green bars in panel A, representing immune-checkpoint molecules and kinases, respectively, have been further broken down in panel B. The orange bar in panel B represents protein tyrosine kinases whose individual members are shown in the bottom right panel. Data from CAS Content Collection over 2020–2022. Targets can be divided into four categories of growth: very fast (>10%), fast (4–10%), modest (1–4%), and slow (<1%).

Out of the ∼100 identified emerging targets, publications for protein targets such as gasdermins,^23^ STING,^24^ cell division cycle protein 20 (CDC20),^25^ trophoblast cell-surface antigen 2 (TROP2),^26^ proprotein convertase subtilisin/kexin type 9 (PCSK9),^27^ leucocyte immunoglobulin like receptor B1 (LILRB1),^28^ and cyclin-dependent kinases (CDK1^30^ and CDK4/6^31^) have doubled, while those for nectin-4,^29^ α-enolase 1 (ENO1),^30^ and hematopoietic progenitor kinase (HPK)^31^ have nearly tripled over the past three years (2020–2022). Identified therapeutic targets span a wide spectrum, ranging from those with obvious connections to those that might be more obscurely related to cancer immunotherapy nonetheless being increasingly investigated in the context of cancer immunotherapy (KRAS, kinase inhibitors etc.). Concepts/areas of interest where the relative growth rates of publications are high despite low absolute number of publications might indicate burgeoning and unmet interest (Figure 4, Figures S2–S4)—potential examples include gasdermins, TROP2, and nectin-4, all of which are among the fastest growing concepts, with relative growth rates averaging >10% over the past three years (Figures 2 and 3). In contrast, for concepts such as immune checkpoint molecules, kinases, and glycoproteins, the gap between pace of growth and absolute number of publications is not as stark, indicating that these concepts might be at the later stages of emergence (Figure 4A). For about half of the identified emerging targets, the number of publications increased by 1.1–1.5× (moderate pace); a few representative ones are human epidermal growth factor receptor 2 (HER2),^32^ toll-like receptors (TLRs),^33^ and mammalian target of rapamycin (mTOR)^34^ (Figures 2 and 4A). A few targets appear to grow at a slow pace, such as cyclooxygenase-2 (COX-2)^35^ (Figure 2).

Delving deeper into inhibitory immune checkpoint molecules, publications for T cell immunoreceptor with Ig and ITIM domains (TIGIT)^36^ and B7-H3^37^ grew on an average 1.5× over the past three years (Figure 2), and this type might be emerging rapidly as compared to the rest of the checkpoint molecules (Figure 4B). PD-1 and programmed cell death ligand 1 (PD-L1)^38^ appear to be growing at a modest pace, while others such as indoleamine 2,3-dioxygenase (IDO) and tryptophan 2,3-dioxygenase (TDO),^39^ enzymes vital for tryptophan degradation, appear to be growing slowly (Figure 2). HPK and TANK-binding kinase 1 (TBK1)^40^ among other kinases are emerging as fast-growing concepts, with publications appearing to have tripled or nearly tripled over the past three years (Figures 2 and 4B). Other members of the kinase family such as janus kinase (JAK)^41^ and pyruvate kinase M2 (PKM2)^42^ are growing at a modest pace (Figures 2 and 4B).

In terms of types of therapies, sonodynamic^43^ and chemodynamic^44^ therapies appear to be emerging at a rapid rate along with RNA-based vaccines, with publications tripling over the past three years (Figure 2). Concepts such as chimeric antigen receptor T-cell (CAR-T) therapy,^45^ nanovaccines,^46^ kinase inhibitors including HPK1^47^ and fms-like tyrosine kinase 3 (FLT3) inhibitors,^48^ as well as STING agonists^49^ and poly-ADP ribose polymerase (PARP) inhibitors,^50^ among others, are also growing at a fairly rapid rate (Figure 2). Examples of therapies growing at a modest pace include anti-HER2 ADCs,^51^ dendritic cell activators,^52^ histone deacetylase (HDAC) inhibitors,^53^ and phosphoinositide 3-kinase (PI3K) inhibitors^54^ (Figure 2).

Overall, targets and biomarkers tend to have a fair degree of overlap, and this is perhaps unsurprising since many biomarkers in cancer end up becoming targets for therapy. This could also be a result of the program picking up phrases outside of a given context (i.e., biomarker vs target), though manual curation of data by subject matter experts has ensured, where possible, that concepts picked out are pertinent. Among biomarkers, nectin-4,^55^ SET domain containing 2 (SETD2),^56^ ENO1,^57^ TROP2,^58^ tumor mutation burden (TMB),^59^ PCSK9,^60^ and LILRB1^61^ appear to be growing at a fast pace, while p62,^62^ COX-2,^63^ and TDO^64^ show unremarkable/negligible growth (Figure 2).

**Figure 5:**
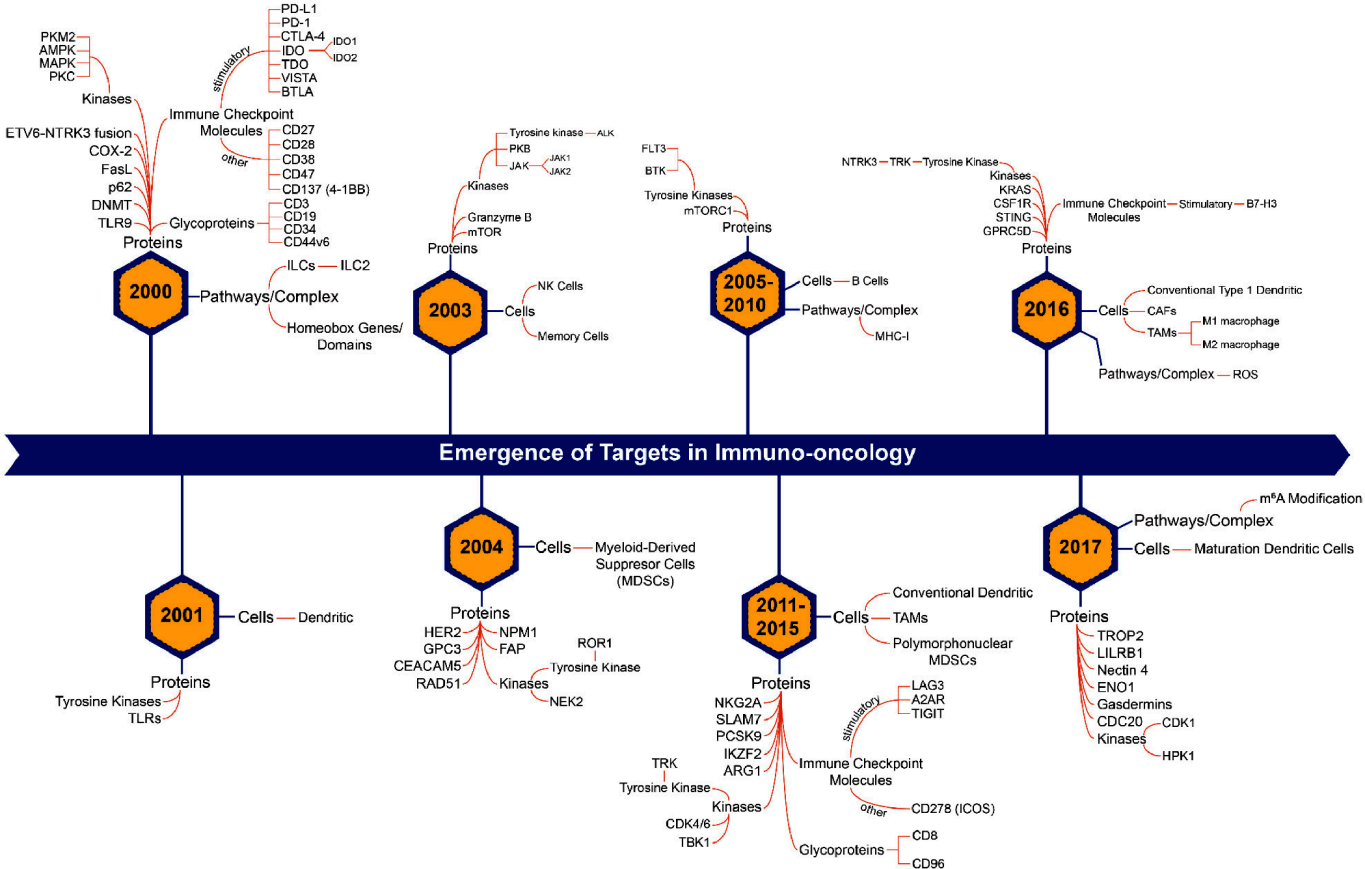
Estimated timeline of emerging therapeutic targets in the field of immuno-oncology based on NLP analysis of >350K publications from the CAS Content Collection for the period 2000–2022.

In addition to the trend landscape map, we also determined the year at which a particular concept started to emerge and crafted timelines for the emergence of targets, therapies, and biomarkers in immuno-oncology (Figures 5, S5, and S6). From the timeline of emerging targets in immuno-oncology (Figure 5), it appears that, while a majority of immune checkpoint molecules emerged in 2000s, others such as lymphocyte-activation gene 3 (LAG3),^65^ adenosine A2A receptor (A2AR),^66^ TIGIT,^67^ and inducible costimulators (ICOS, also known as CD278)^68^ emerged much more recently, between 2011 and 2015. Protein targets such as nectin-4, TROP2, and gasdermins are among the ones that have emerged most recently, in 2017 (Figure 3). Similar timelines for emerging biomarkers and therapies have also been generated (Figures S5 and S6).

## In-Depth View of the Field of Immuno-Oncology: Publication and Substances Trends

We then delved deeper into trends in immuno-oncology covering journal and patent publications, their growth rates as well as co-occurrences between key areas of research.

**Figure 6:**
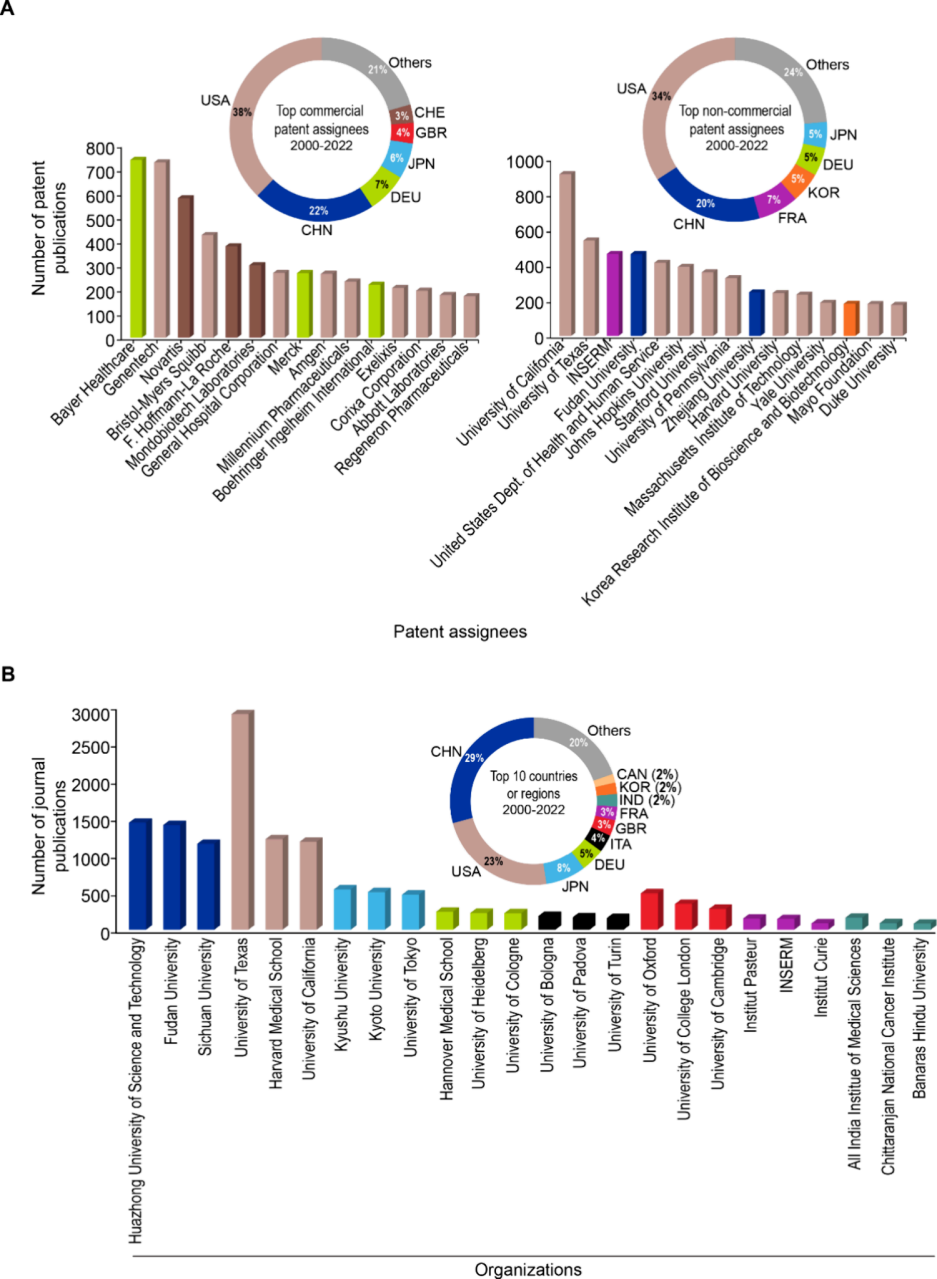
(A) Donut charts indicating distribution of the top 6 countries or regions in terms of patent publications for commercial and noncommercial organizations, while bar graphs show breakdown of the top 15 patent assignee organizations. (B) Donut chart indicates top 10 countries or regions in terms of journal publications for noncommercial organizations, while the bar graph shows the top 3 noncommercial organizations for each of the eight most prolific countries or regions in terms of journal publications. Bar graphs have been colored to match country-specific colors in the donut charts. Data includes publications from the CAS Content Collection for the period 2000–2022.

In patent publications among commercial or noncommercial entities, the Unites States (USA) and China (CHN) dominate, together accounting for nearly 60% and 54% of commercial and noncommercial patent publications, respectively (Figure 6A). Other key players include Germany (DEU), Japan (JPN), Switzerland (CHE), France (FRA), South Korea (KOR), and the United Kingdom (GBR)—though the exact order of prominence varies for commercial vs noncommercial enterprises (Figure 6A). Among commercial patent assignee entities, Bayer Healthcare and Genentech are leaders in terms of the number of patent publications, followed by Novartis, Bristol-Myers Squibb, Roche, and Merck (Figure 6A). Patents held by these commercial organizations appear to be related to monoclonal antibodies, T cells, vaccines, ADCs, CARs, and ICIs, among others.

Like patents, our data show similar trends for journal publications on country-wise distributions, with China and the United States together accounting for 50% of journal publications. The top three leaders in journal publications among the eight leading countries are highlighted in Figure 6B. Within China, Huazhong University of Science and Technology, Fudan University, and Sichuan University lead. Meanwhile, in the United States, the University of Texas, Harvard Medical School, and the University of California are the leaders (Figure 6B).

**Figure 7:**
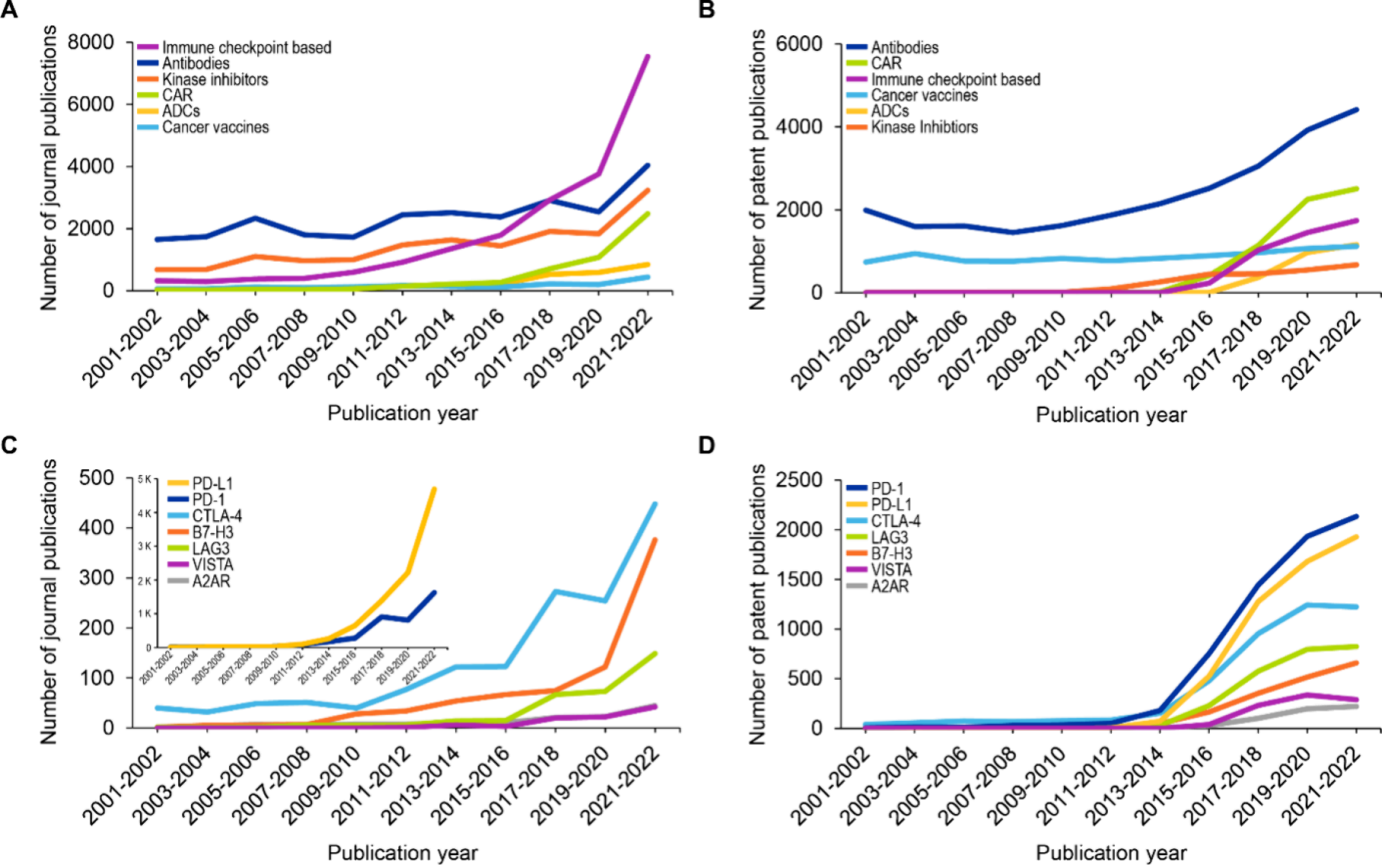
Growth of selected scientific concepts over the past two decades in terms of number of publications. (A) Journal and (B) patent publication trends of selected therapy-related concepts and (C) journal and (D) patent publication trends of selected individual immune checkpoint molecules. Data includes publications from the CAS Content Collection for the period 2001–2022.

To gain insights into how different types of therapies have been investigated over the years, we analyzed the trends of selected emerging concepts that have shown commercial success in cancer immunotherapy, such as immune checkpoint-based therapies, CARs, ADCs, etc. The results indicate an upward trend for all the selected concepts, with a steady growth in number of journal and patent publications over the past two decades (Figure 7). Of special note are immune checkpoint-based therapies, growing sharply after 2015 and nearly doubling between 2020 and 2022 (Figure 7A,B). Since immune checkpoint-based therapy has been rising faster than others, we looked at the growth trajectory of individual checkpoint proteins. Within immune checkpoint molecules, PD-L1 and PD-1 show the greatest number of journal and patent publications, with PD-L1 having nearly twice as many journal publications as all the other immune checkpoint molecules put together in 2022 (Figure 7C). While PD-1 and PD-L1 dominate in terms of absolute number of publications, when looked at from the lens of relative growth, other immune checkpoint molecules, such as B7-H3, LAG3, and V-domain immunoglobulin suppressor of T cell activation (VISTA), appear to be keeping pace with PD-1 and PD-L1 (Figure S7).

**Figure 8:**
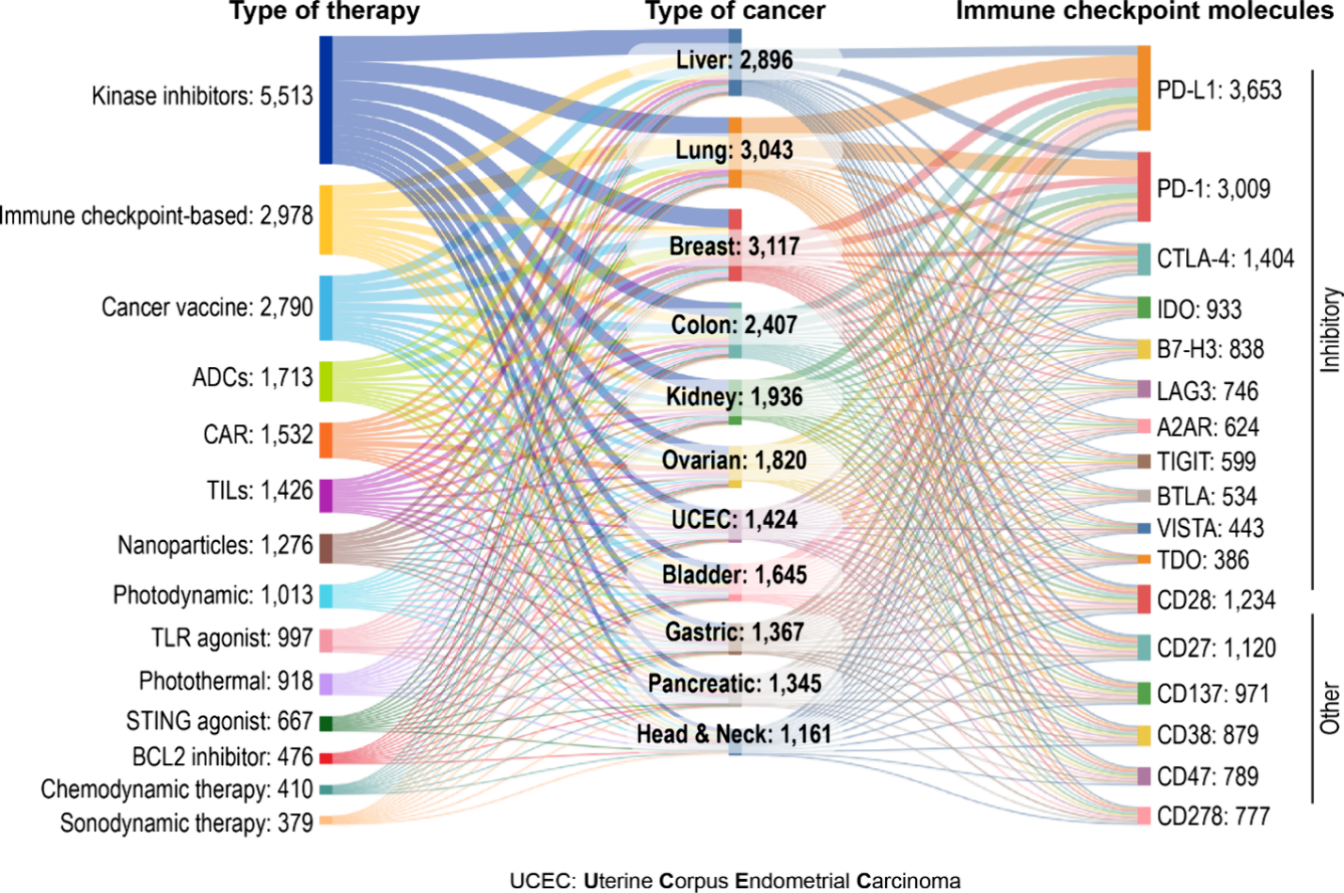
Sankey plot indicating co-occurrences of therapies (left) and types of cancer (center), and types of cancer (center) with specific immune checkpoint molecules (right), in journal publications. Data includes journal publications from the CAS Content Collection for the period 2000–2022. Abbreviations: ADC, Antibody–Drug Conjugate; CAR, Chimeric Antigen Receptor; TIL, Tumor Infiltrating Lymphocyte; TLR, Toll-like Receptor; BCL2, B-cell Lymphoma 2; UCEC, Uterine Corpus Endometrial Carcinoma.

To illustrate the connection between emerging therapies and different types of cancer, we determined instances of co-occurrences among related concepts, as shown in Sankey graphs (Figure 8). Among the different types of emerging therapies, kinase inhibitors, immune checkpoint-based therapies, cancer vaccines, ADCs, CAR, and tumor infiltrating lymphocytes (TILs) appear to co-occur to a larger extent with emerging solid tumors in journal publications related to immuno-oncology (Figure 8). Key observations/takeaways from this co-occurrence analysis (Figure 8) are as follows:

- Immune checkpoint-based therapies appear to co-occur the most with lung cancer, followed by comparable co-occurrences with liver, breast, colon, and kidney cancer, and to the least extent with head and neck cancer. Both PD-1 and PD-L1 co-occur twice as many times with lung cancer as compared to breast cancer, the second most co-occurring type of cancer (Figure 8). This high co-occurrence can be attributed to the discovery of PD-1/PD-L1 inhibitors as effective treatment for NSCLC.^69^ Cytotoxic T-lymphocyte-associated antigen 4 (CTLA-4) shows a slightly higher degree of overlap with lung cancer as compared to other solid tumors. Other inhibitory immune checkpoint molecules, such as IDO, B7-H3, LAG3, A2AR, TIGIT, B and T lymphocyte attenuator (BTLA), VISTA, and TDO, appear to have similar co-occurrences with the identified emerging solid cancer types. Other immune checkpoint molecules (sometimes considered stimulatory), such as CD27, CD38, CD47, CD137, and CD278 (Figure 8), exhibited similar degrees of co-occurrences with emerging solid tumors. Immune checkpoint molecules (both inhibitory and others/stimulatory) show no particular preferences for liquid tumors, except for CD38 with multiple myeloma (MM) and acute myeloid leukemia (AML) (Figure S8).
- Unsurprisingly, ADCs show the greatest overlap with breast cancer (Figure 8), and this can be attributed to the development and approval of ado-trastuzumab emtansine (T-DM1/Kadcyla),^70^ trastuzumab deruxtecan (T-DXd/Enhertu),^71^ and sacituzumab govetican (SG/Trodelvy)^72^ for the treatment of breast cancer. This is followed closely by co-occurrences of ADCs with lung cancer indicating increased traction.^73^ Trastuzumab deruxtecan (DS-8201a, T-DXd/Enhertu) became the first ADC to gain U.S. FDA approval for the treatment of lung cancer in 2022.^74^
- Cancer vaccines co-occur more with liver, lung, breast, and colon cancer as compared to other types of cancer.
- Kinase inhibitors co-occur with lung, liver, and breast cancer over most of the other types (Figure 8).
- CARs show the highest co-occurrence with ovarian cancer. While originally most effective against hematological malignancies, in recent years there has been an increasing push toward the use of CAR-T for solid tumors, with a lot of research focused around ovarian cancer, perhaps accounting for this trend.^75^
- TLR agonists, B-cell lymphoma 2 (BCL2) inhibitors, and STING agonists display similar degrees of co-occurrences with various types of solid tumors.
- Pharmaceutical nanoparticles, often utilized to deliver anticancer drugs, appear to show a slightly higher degree of overlap with breast cancer as compared to the rest.
- Therapies such as photodynamic, photothermal, sonodynamic, and chemodynamic appear to show no particular preference for types of cancer.
- In terms of liquid tumors, almost all the selected therapies show equal co-occurrences with the 4 types of emerging liquid tumors except for cancer vaccine, which appears to show a slight preference for AML and MM as compared to ALL and diffuse large B-cell lymphoma (DLBCL) and kinase inhibitors for ALL and AML (Figure S8).

**Figure 9:**
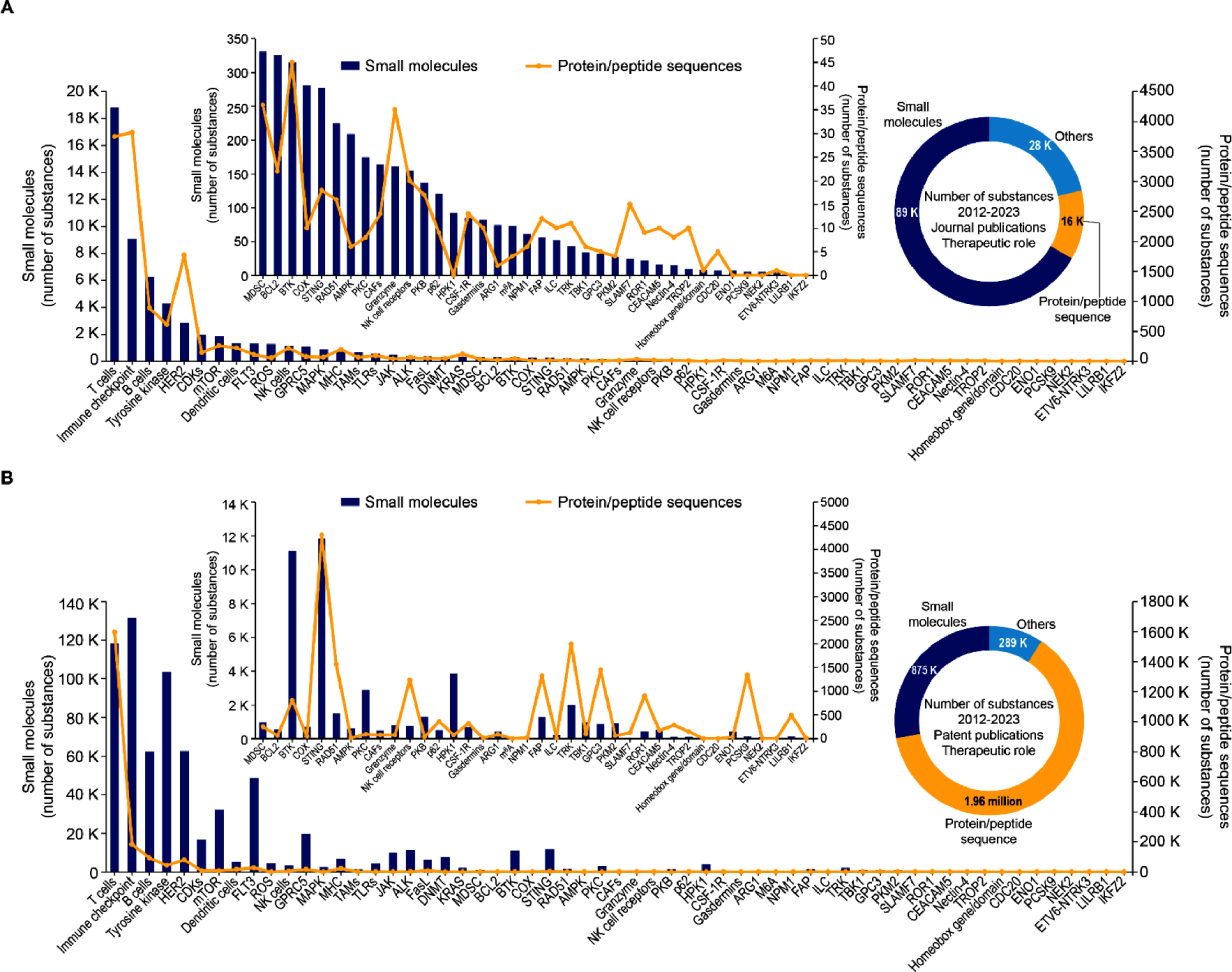
Trends from substance data analysis on data retrieved from CAS Registry associated with selected emerging targets in (A) journal and (B) patent publications for the period 2012–2022, focused on the two major substance classes: small molecules (blue bars) and protein/peptide sequences (yellow line). Inset donut charts depict overall distribution across substance classes. Only substances indexed as therapeutic (THU) were included in the analysis.

Our substance database, CAS Registry,^76^ consists of >219 million substances indexed based on a host of information including substance roles (>35 unique roles), substance classes, including small molecules, protein/peptide sequences, polymers, nucleic acids, etc., CAS index terms, and CAS registry numbers. Listed below are our observations from analysis of data from >3.2 million substances in the field of immuno-oncology for the period 2012–2022:

- On the whole, the number of substances associated with patent publications (∼3.1 million) is significantly higher than those associated with journal publications (∼133K), being over 20× higher (Figure 9).
- Therapeutic substances of both small molecules and protein/peptide sequences associated with journal publications in the field of immuno-oncology show a steady growth since 2012, with a sharp increase in small molecules post 2020 (Figure S9A).
- For therapeutic substances associated with patent publications, protein/peptide sequence of substance classes are 2× that of small molecules (Figure 9B donut chart), suggestive of greater commercial interest in development of cancer immunotherapeutics with proteins/peptides.
- Co-occurrence analysis of substances with specific emerging concepts using CAS indexed terms, with an emphasis on small molecule and protein/peptide sequences (Figure 9), indicate the following:
  - Therapeutic targets such as T cells, immune checkpoint molecules, B cells, tyrosine kinase, and HER2 appear to lead across both patent and journal publications.
  - For journal publications: (1) Therapeutic targets of low co-occurring frequency (<100) with substances include HPK, colony-stimulating factor 1 receptor (CSF-1R), arginase 1 (ARG1), nucleophosmin 1 (NPM1), fibroblast activation protein (FAP), innate lymphoid cells (ILC), TBK1, glypican-3 (GPC3), carcinoembryonic antigen cell adhesion molecule 5 (CEACAM5), nectin-4, TROP2, and CDC20, among a few others (Figure 9). (2) Therapeutic targets exhibiting sharp upward growth trends (most noticeably in 2022) in co-occurring frequency with substances include dendritic cells, cancer-associated fibroblasts (CAFs), tumor-associated macrophages (TAMs), STING, gasdermins, CDC20, TROP2, LILRB1, TBK1, PKM2, protein kinase B (PKB), NIMA-related kinase 2 (NEK2), and ARG1.
  - For patent publications: While the overall number of protein/peptide sequences is 2× that of small molecules, this phenomenon was not uniform across identified emerging therapeutic targets. The protein/peptide sequence to small molecule ratio sheds light on possible commercial interests (Figure S10). For instance, therapeutic targets such as T cells, PCSK9, dendritic cells, major histocompatibility complex (MHC), and LILRB1 show an overwhelmingly high co-occurrence with protein/peptide sequences. Targets such as RAD51, FAP, and receptor tyrosine kinase (TRK) co-occur with protein/peptide sequences and small molecules to an equivalent extent.

## Current Overview of Immuno-Oncological Therapeutics in Development

Data retrieved from Pharmaprojects^77^ about drugs currently in the developmental pipeline indicates a steady increase since 2012 (Figure S11A), with a vast majority of drugs in preclinical stages or early stages of clinical trials (phases I and II). These drugs are distributed across a wide range of therapeutic classes such as antibodies, gene therapy, vaccines, etc. (Figure S11B). The United States and China are the leaders in this developmental effort, with a majority of the leading companies that are currently conducting research in immuno-oncology based in the U.S. or China (Figure S11C). Key players in this commercial-driven research are highlighted in Figure S11C and include well-known pharmaceutical companies such as Bristol-Myers Squibb, Roche, Merck, Sanofi, and AstraZeneca, among others. Areas of interest include protein targets such as immune checkpoint molecules, interleukins, kinases, and chemokines and cellular targets such as T cells and natural killer (NK) cells (Figure S11D).

## Therapies in the Development Pipeline

### Immune Checkpoint Molecules: Continued Interest in Development of Drugs

Cancer cells employ various strategies such as attracting regulatory T (T_reg_) cells, reducing the expression of tumor antigens, triggering T cell tolerance or apoptosis, and generating immune-suppressing cytokines. The immune-suppressing cytokines may activate inhibitory immune checkpoints, resulting in a high immunosuppressive tumor microenvironment (TME).^78^ Immune checkpoint molecules balance between pro-inflammatory and anti-inflammatory signaling, functioning as key regulators of immune responses to aid the immune system in recognition and elimination of malignant cells. They are further classified into inhibitory or stimulatory/other checkpoint molecules (Figure 2).

ICIs such as monoclonal antibodies work by blocking the effects of inhibitory pathways and allow T cell activation. ICIs targeting CTLA4, PD-1, PD-L1, and LAG3 have been approved by the U.S. FDA since 2011 (Table 1), totaling 11 compounds, four of which were approved in the past 2 years. Anti-CTLA-4, anti-PD-1, anti-PD-L1, and anti-LAG3 agents are utilized in the treatment of multiple solid and hematologic malignancies; however, one of their biggest caveats is that their effectiveness is restricted to only a specific subset of patients. The newest approved category, anti-LAG3 agents, received their first U.S. FDA approval in March 2022. Opdualag, a fixed-dose combination of the LAG3 blocking antibody relatlimab and the PD-L1 inhibitor antibody nivolumab, has been approved for the treatment of unresectable or metastatic melanoma.^79^

**Table 1:** Selected U.S. FDA-Approved Immune Checkpoint Inhibitors, CAR-T Cell Therapies, Antibody–Drug Conjugates, and Cancer Vaccines^a^

**Name**	**Target/Type**	**Year of Approval**	**Company**	**Disease Indication**	**CAS REG Number**
**Immune Checkpoint Inhibitors (ICIs)**					
Ipilimumab/Yervoy	CTLA-4	2011	Bristol-Myers Squibb	Melanoma, RCC, CRC, HCC, NSCLC, MPM, EC	477202-00-9
Tremelimumab/Imjudo	CTLA-4	2022	AstraZeneca	HCC	745013-59-6
Pembrolizumab/Keytruda	PD-1	2014	Merck	Melanoma, NSCLC, HNSCC, cHL, PMBCL, urothelial carcinoma, CRC, gastric cancer, EC, CC, HCC, MCC, RCC, endometrial cancer, TMB-H cancer, cSCC, TNBC, PMBCL	1374853-91-4
Dostarlimab/Jemperli	PD-1	2021	GlaxoSmithKline	Endometrial cancer, dMMR solid tumors	2022215-59-2
Retifanlimab/Zynyz	PD-1	2023	Incyte	MCC	2079108-44-2
Durvalumab/Imfinzi	PD-L1	2017	AstraZeneca	NSCLC, SCLC, BTC, HCC	1428935-60-7
Avelumab/Bavencio	PD-L1	2017	EMD Serono	Urothelial carcinoma, MCC	1537032-82-8
Nivolumab and Relatlimab/Opdualag	PD-1/LAG3	2022	Bristol-Myers Squibb	Melanoma	2769698-54-4

**CAR-T Cell Therapy**					
Tisagenlecleucel/Kymriah	CD19	2017	Novartis	B-cell ALL, B-cell NHL	1823078-37-0
Axicabtagene ciloleucel/Yescarta	CD19	2017	Kite Pharma/Gilead	B-cell NHL, Follicular lymphoma	2086142-87-0
Brexucabtagene autoleucel/Tecartus	CD19	2020	Kite Pharma/Gilead	B-cell ALL, MCL	2691112-12-4
Lisocabtagene maraleucel/Breyanzi	CD19	2021	Juno Therapeutics, Bristol-Myers Squibb	B-cell NHL	2099722-39-9
Idecabtagene vicleucel/Abecma	BCMA	2021	Celgene Corporation, Bristol-Myers Squibb	MM	2306267-75-2
Ciltacabtagene autoleucel/Carvykti	BCMA	2022	Janssen Biotech	MM	2641066-71-7

**Antibody–Drug Conjugates (ADCs)**					
Trastuzumab emtansine/Kadcyla	HER2	2013	Genentech, Roche	Metastatic HER2-positive breast cancer	1018448-65-1
Inotuzumab ozogamicin/Besponsa	CD22	2017	Pfizer	CD22-positive ALL	635715-01-4
Loncastuximab tesirine-lpyl/Zynlonta	CD19	2021	ADC Therapeutics	Large B-cell lymphoma	1879918-31-6
Tisotumab vedotin-tftv/Tivdak	Tissue Factor	2021	Seagen	Recurrent or metastatic CC	1418731-10-8
Mirvetuximab soravtansine/Elahere	FRα	2022	ImmunoGen	Platinum-resistant ovarian cancer	1453084-37-1

**Cancer Vaccines**					
BCG Live	Fibronectin receptors	1990	Organon Teknika Corp.	Bladder cancer	2223648-65-3
Oncophage/Vitespen	Heat shock protein peptide complex HSPPC-96-based vaccine	2008 (approved in Russia)	Antigenics (now Agenus)	Kidney cancer, metastatic melanoma, and glioma	492448-75-6
Provenge/Sipuleucel-T	Dendritic cell vaccine	2010	Dendreon Pharmaceuticals	Asymptomatic or minimally symptomatic HRPC	917381-47-6
Talimogene laherparepvec/T-VEC/Imlygic	Live attenuated HSV1 virus	2015	Amgen, BioVex	Stage IIIb-IVM1c melanoma	1187560-31-1

a Abbreviations: ALL, **A**cute **L**ymphoblastic **L**eukemia; BTC, **B**iliary **T**ract **C**ancer; CC, **C**ervical **C**ancer; cHL, **C**lassical **H**odgkin’s **L**ymphoma; CRC, **C**olo**r**ectal **C**ancer; cSCC, **C**utaneous **S**quamous **C**ell **C**arcinoma; dMMR, **d**eficient **m**is**m**atch **r**epair; EC, **E**sophageal **C**ancer; HCC, **H**epatocellular **C**arcinoma; HNSCC, **H**ead and **N**eck **S**quamous **C**ell **C**ancer; HRPC, **H**ormone-**R**efractory **P**rostate **C**ancer; MCC, **M**erkel **C**ell **C**arcinoma; MCL, **M**antle **C**ell **L**ymphoma; MPM, **M**alignant **P**leural **M**esothelioma; MM, **M**ultiple **M**yeloma; NHL, **N**on-**H**odgkin’s **L**ymphoma; NSCLC, **N**on-**S**mall-**C**ell **L**ung **C**ancer; PMBCL, **P**rimary **M**ediastinal **L**arge **B**-cell **L**ymphoma; RCC, **R**enal **C**ell **C**arcinoma; SCLC, **S**mall-**C**ell **L**ung **C**ancer; TMB-H, **T**umor **M**utational **B**urden-**H**igh; TNBC, **T**riple **N**egative **B**reast **C**ancer.

Stimulating ICs hold great therapeutic promise—urelumab and utomilumab (PF-05082566) are two anti-CD137 antibodies under development^80^ that appear promising in renal cell carcinoma and colon cancer among many others because they can stimulate cytotoxic T cells and enhance the production of interferon gamma (IFN-γ), which is crucial for anticancer effects. CD38 plays a role in suppressing immune responses mediated by IFNγ^81^ and is expressed on M1 macrophages, as well as in immune responses involving neutrophils and T cells. Daratumumab (Darzalex, Janssen Biotech Inc.),^82^ an anti-CD38 IgG1 antibody specific to humans, has received U.S. FDA approval^83^ for use either on its own or in combination therapies to treat relapsed or refractory MM^84^ and is effective at inducing both complement-dependent cytotoxicity and antibody-dependent cell-mediated cytotoxicity against MM cells.^85^

### CAR-T Therapy: Important Cellular Immunotherapy

Chimeric antigen receptor T cells, or CAR-T cells—engineered cytotoxic T cells that express synthetic CAR receptors^86^—have been of continued interest to the scientific community since their onset. Our trend landscape analysis (Figure 2) indicates continued growth of publications for CAR therapies (CAR-T, CD19 CAR, CAR-NK). Chimeric receptors combine antigen recognition and T cell binding capability in a single receptor. The receptor portion of CARs consists of four prominent segments—an extracellular domain derived from the single-chain variable fragment (sc-Fv) of the antibody, which is responsible for tumor antigen recognition, a hinge region that connects the sc-Fv fragment to the transmembrane (TM) domain, and the intracellular signaling domain. The hinge region and the TM domain anchor CARs to the cell membrane and help in the downstream signaling cascade for T cell activation.^87^

CAR-T cells are created by harvesting T cells from the patient’s blood, genetically modifying them to target cancer cells specifically, and multiplying their numbers. These modified cells are then reintroduced into the patient’s bloodstream to attack the cancer cells. CAR-T cells have shown promising results in treating hematological cancers, such as ALL, non-Hodgkin’s lymphoma (NHL), and MM.^88,89^ Kymriah (tisagenlecleucel) was the first U.S. FDA-approved CAR-T cell therapy, developed by Novartis.^90^ This CD19-directed therapy was approved in 2017 for the treatment of B-cell ALL. To date, five more CAR-T cell therapies have been approved by the U.S. FDA (Table 1). While CAR-T cell therapy has shown remarkable success in treating hematological cancers,^91^ their use in solid tumors such as gastric,^92^ pancreatic,^93^ and lung cancer^94^ is still in exploratory stages. The challenge of using CAR-T immunotherapy in solid tumors arises from a lack of suitable CAR-T antigens and the tumors’ immune-suppressive and complex microenvironment, which poses difficulties in the trafficking of CAR-T cells.^95^ Current research is focused on improving infiltration of solid tumors by administering CAR-T-based therapy directly to the tumor site rather than through the bloodstream. To date, >1,100 trials for CAR-T therapies are ongoing,^96^ of which ∼150 are directed toward solid tumors.^97^ In recent years, CAR-NK and CAR-M therapies have been developed as alternate/synergistic therapies for solid tumors.^98^

Despite their effectiveness, CAR-T therapies have disadvantages, such as heightened immune reactions including cytokine release syndrome, CAR T cell-related encephalopathy syndrome, off-tumor toxicities, and immune effector cell-associated neurotoxicity syndrome.^88,99^ Another major concern is the high cost of therapy. However, various efforts are being made to counter the drawbacks of CAR-T therapy, such as optimizing autologous CAR-Ts for liquid tumors,^100^ expanding CAR administering facilities, and developing next-generation CAR-T therapies.

### Antibody–Drug Conjugates (ADCs): Effective and Promising “Magic Bullets”

ADCs are among the most promising drug classes in oncology currently, with the development accelerating in the past 20 years. An ADC is composed of a monoclonal antibody and a cytotoxic payload connected with a linker to create a targeted immunoconjugate.^15^ Despite being proposed in the early 1900s by Paul Ehrlich as a “magic bullet” that targets pathogens,^101^ it was not until the 1980s that ADCs were first explored in clinical trials, with the first ADC gaining U.S. FDA approval in the year 2000.

Optimization and selection of antibodies, linkers, and payloads helped fuel accelerated ADC development. Ideal antibody characteristics include selective binding and high affinity for the target antigen, a long half-life, low immunogenicity, and superior linker binding.^102^ Currently, ADCs widely use the immunoglobulin G1 antibody.^103^ Recent advances in antibody engineering research have offered new alternatives and include bispecific and trispecific antibodies targeting two and three distinct antigens^104^ and also nanobodies, single domain antibody fragments.^105^ The linker moiety of an ADC should be stable in plasma, not alter the characteristics of the antibody or drug, be hydrophilic to mitigate lipophilic payload solubility effects, and release the payload selectivity and completely.^106^ They are divided into two major classes, cleavable and noncleavable, with cleavable linkers dominating both approved ADCs and ADCs currently in clinical trials.^107^ Lastly, the payload used for ADCs must also be stable in both blood and plasma and needs to be highly potent in small doses due to their selective release.^108^ A range of payloads are currently being used in approved ADCs, including suristatins, calicheamicin, duocarmycins, maytansinoids, and pyrrolobenzodiazepines, with payloads such as amberstatin, eribulin, and tubulysin currently researched in clinical trials.^109^

Over 150 unique ADC candidates are being researched in clinical trials, with their most utilized target antigens being HER2, followed by TROP2, Claudin 18.2, cMET, and B7-H3, respectively. Eight unique bispecific ADCs have also entered clinical trials for the treatment of various solid tumors.^15^ Among the various payloads utilized, topoisomerase I inhibitors, which impact DNA replication in cancer cells, are currently utilized by over 40 ADC candidates in clinical trials. Fifteen ADCs have gained regulatory approval across a wide range of cancer types, including solid tumors such as breast, gastric, and ovarian as well as hematologic malignancies such as lymphoma and leukemia.^15^Table 1 showcases a few promising regulatory approved ADCs to highlight the diversity among target antigens, disease indications, companies, and regulatory approval dates.

### Cancer Vaccines: Beneficial Preventive Strategy

Cancer vaccines are a form of immunotherapy aimed at harnessing the body’s immune system to fight against cancer cells by reprogramming the immune system and activating a T cell-mediated adaptive immune response to attack “foreign” cancer cells.^110^ Cancer vaccines are capable of targeting either tumor surface antigens (TSAs) or tumor-associated antigens (TAAs). TSAs are proteins unique to cancer cells that are not expressed in healthy cells,^110,111^ while TAAs, on the other hand, are expressed in normal as well as cancer cells to differing extents—higher expression levels in cancer cells make them suitable targets for developing cancer vaccines.^112^ Cancer vaccines can be administered either as “preventive” aids or as “therapeutics” to prevent the occurrence of tumors or cure the disease, respectively. Examples of the former include Cervarix (GlaxoSmithKline Biologicals),^113^ Gardasil-4 (Merck & Co.), Gardasil-9 (Merck Sharp & Dohme LLC),^114^ and Hepatitis B (HBV) vaccine (HEPLISAV-B, Dynavax Technologies Corporation);^115^ examples of the latter include Bacillus Calmette-Guerin (BCG)^116^ and Provenge (sipuleucel-T; Dendreon Pharmaceuticals, LLC).^117^Table 1 highlights a few selected cancer vaccines with respect to their targets, disease indications, companies, and regulatory approval dates.

Below are a few types of therapeutic cancer vaccines depending on the design strategy and the mode of action:

1. **Peptide vaccines** – These vaccines contain small fragments of cancer-specific peptides derived from either TSA- or TAA-containing T cell epitopes.^118^ When injected into the body, they elicit an immune response that targets cancer cells expressing these peptides, e.g., HBV and HPV vaccines.
2. **Whole-cell vaccines** – Whole-cell vaccines use whole tumor cells or cell lysates typically modified/treated to enhance their ability to trigger an immune response, aimed to elicit stronger and more diverse immune reaction^119^ by presenting a broad range of tumor antigens. Provenge (sipuleucel-T; Dendreon Pharmaceuticals, LLC)^117^ is the first U.S. FDA-approved dendritic cell vaccine, a type of whole-cell vaccine.^120^
3. **Virus-based/Oncolytic vaccines** – Viruses, engineered to carry cancer-specific antigens, trigger immune response against cancer cells expressing those antigens upon administration.^121,122^ Nadofaragene firadenovec-vncg (Adstiladrin; Ferring Pharmaceuticals A/S)^123^ is a 2022 U.S. FDA-approved non-replicating adenoviral vector-based therapy for treating adults with high-risk BCG unresponsive non-muscle-invasive bladder cancer with carcinoma in situ with/without papillary tumors.^124^ Oncolytic viral therapy relies on viruses that kill cancer cells without harming normal cells^122^ and is the basis for the development of the U.S. FDA-approved talimogene laherparepvec (T-VEC or Imlygic; BioVex Inc.)^125^ used for treatment of melanoma utilizing herpes simplex virus type 1.
4. **Nucleic acid vaccines** – Genetic material (DNA) encoding specific cancer antigens is introduced into the patient’s cells, leading to expression of said antigens eliciting an immune response against the cancer. DNA vaccines for cancer immunotherapy are under clinical trials (e.g., NCT04090528, NCT03199040, and NCT04251117). mRNA-based vaccines are currently in development and are undergoing clinical trials (e.g., NCT04382898 and NCT03164772), gaining importance in cancer immunotherapy.^126^ Nucleic acid vaccines are safe, low/non-immunogenic, potent, fast acting, and easier to manufacture.^127^
5. **Nanovaccines** – Nanocarriers encapsulating antigens, thereby preventing their degradation, are being used to treat cancers and infectious diseases such as COVID-19. Given their small size, the use of adjuvants along with target antigens helps in their successful delivery to antigen-presenting cells.^128^

A closer look at our data indicates that the number of publications related to cancer vaccines in immunotherapy has grown at a modest rate over 2020–2022 (Figures 2 and 7). Among the various types of cancer vaccines, RNA-based and nanovaccines have grown the fastest in the past three years (Figure 2).

### Natural Killer (NK) Cells: Promising Cancer Immunotherapeutics

NK cells, a type of cytotoxic lymphocyte, play a critical role in the immune system’s surveillance and elimination of cancer cells. Given their potent antitumor activity, NK cells are being actively explored as viable targets in cancer immunotherapy in several ways:

1. **Adoptive cell therapy (ACT)** – ACT involves isolating and expanding NK cells from a patient’s own blood (autologous) or from a donor (allogenic), followed by their infusion back into the patient.^129,130^ The initial use of NK cells in treating cancer involved infusing IL-2-activated cells into patients in the 1980s.^131^ However, efficacy was limited due to IL-2 toxicity and unknown factors such as the impact of T_reg_ cells. Later, evidence in 2002 indicated the clinical benefit of NK cells in patients receiving bone marrow transplants, showing lower relapse rates in certain conditions.^132^ Various trials involving adoptive cell transfer of activated NK cells demonstrated some success, especially when there was evidence of the cells persisting and expanding post infusion.^130,133^ Recent studies have shown promise in enhancing NK cell efficiency by preactivating them with a combination of cytokines, resulting in improved functions both *in vitro* and *in vivo* in mouse models of cancer.^134^
2. **Engineered NK cells** – Recent research has shown achievements through the use of engineered NK cells containing activating chimeric antigen receptors (CARs) designed for targeting tumor-specific antigens.^135^ When CAR-NK cells developed from umbilical cord blood transduced with a modified retroviral vector expressing genes for an inducible caspase-9 suicide switch, an anti-CD19 CAR, and IL-15 were used to treat patients with CD19+ conditions, they exhibited favorable expansion, long-lasting presence, and positive outcomes, with no significant adverse effects observed.^136^
3. **Combination therapies** – NK cell-based therapies are often used in combination with other immunotherapies, such as ICIs or monoclonal antibodies, to enhance their efficacy^137^ by overcoming tumor immunosuppression and promoting more robust antitumor immune responses. Typical immune checkpoint PD-1/CTLA-4 inhibitors have been used not only to relieve the inhibitory state of T cells but also to reverse the incapacity of NK cell.^138^
4. **Targeting inhibitory receptors** – Strategies are being explored to block or downregulate inhibitory receptors on NK cells, such as NKG2A, to overcome tumor-induced immunosuppression and enhance NK cell activity.^139^

Despite their great promise, challenges remain in NK cell-based immunotherapy, including optimizing NK cell expansion and persistence, improving homing to tumor sites, and overcoming immunosuppressive tumor microenvironments. Ongoing research and clinical trials are aimed at addressing these challenges and further unlocking the therapeutic potential of NK cells in cancer treatment. A few of these clinical trials are listed in Table 2 to show a snapshot of this currently active clinical trial landscape.

**Table 2:** Selected Clinical Trials Involving NK Cell-Based Therapy^a^

**NK Cell Strategy**	**Indication**	**Intervention**	**Status**	**Sponsor**	**Clinical Trial ID**
Engineered NK cells	Ovarian cancer	Natural killer Group 2D CAR-NK Cells	Recruiting	Hangzhou Cheetah Cell Therapeutics	NCT05776355
Combination therapy	HER2 breast cancer	Allogenic NK cells and Trastuzumab and Pertuzumab	Recruiting	Vall d’Hebron Institute of Oncology	NCT05385705
Combination therapy	Neuroblastoma	Hu3F8 (Anti-GD2 antibody) and allogeneic NK cells	Active	Memorial Sloan Kettering Cancer Center	NCT02650648
Targeting inhibitory receptors and combination therapy	HL or NHL	AFM13-NK and AFM13 monoclonal antibody	Active	M.D. Anderson Cancer Center	NCT04074746

a Abbreviations: HL, **H**odgkin’s **L**ymphoma; NHL, **N**on-**H**odgkin’s **L**ymphoma.

### Tumor-Associated Macrophages (TAMs): Reprogramming the Tumor Microenvironment

TAMs, a type of immune cells that infiltrate the TME and play a crucial role in tumor progression and immunosuppression, are derived from circulating monocytes that are recruited to the tumor site by various chemokines and cytokines produced by the tumor cells.^140^ Generally, TAMs can be classified into two main phenotypes: M1 and M2.^141^ The M1 phenotype, also known as classically activated macrophages, is associated with pro-inflammatory responses and antitumor activity. In contrast, the M2 phenotype, also called alternatively activated macrophages, exhibits anti-inflammatory properties and promotes tumor growth, angiogenesis, tissue remodeling, and immunosuppression.^141^ Listed below are ways in which TAMs are currently being explored in cancer immunotherapy:

1. **CAR-M cells** – Engineered cells with edited CARs to identify specific antigens on cancer cells, enhancing their ability to recognize and engulf these cells by presenting tumor antigens to Th1 cells, leading to the production of anti-inflammatory factors, which in turn activate T-cell-mediated immunity. Carisma Therapeutics is currently recruiting patients for an early phase I study of their engineered HER2-targeted CAR-M cell therapeutic CT-0508 (NCT04660929). CT-0508 will be studied in combination with the ICI pembrolizumab for the treatment of HER2-overexpressing solid tumors.^142^
2. **Repolarization of TAMs** – The goal is to shift TAMs from the M2 phenotype to the M1 phenotype, exploiting the pro-inflammatory responses and antitumor activity of M1 macrophages, and can be achieved by using specific molecules or drugs that modulate the macrophage polarization state,^143^ thereby promoting antitumor immune responses.^144^
3. **Depletion of TAMs** – Reducing the number of TAMs within the tumor microenvironment by targeting specific surface markers or by blocking the recruitment of monocytes to the tumor site appears to be a potentially viable approach.^145^ This strategy is being studied for the treatment of platinum-resistant epithelial ovarian cancer (NCT05053750). The TAM-targeting therapeutic, zoledronic acid, a bisphosphonate drug, blocks the mevalonate pathway, causing macrophage apoptosis and depletion^146^ in combination with paclitaxel and bevacizumab.
4. **Inhibition of TAM-associated signaling pathways** – TAMs can secrete various immunosuppressive factors and cytokines that promote tumor growth and immunosuppression. Targeting these signaling pathways, such as CSF-1R, can inhibit TAM recruitment and function.^147^ The M.D. Anderson Cancer Center is currently researching this strategy, consisting of a combination of TAM depletion and inhibition of TAM-associated signaling in the above-mentioned active early phase I clinical trial NCT05053750.

By targeting TAMs, researchers aim to reprogram the tumor microenvironment to promote an immune-activating and tumor-suppressing environment. However, it is important to note that TAMs’ role in cancer is complex and context-dependent, and further research is needed to better understand their diverse functions and develop effective TAM-targeted therapies.

## Modes of Delivery: Optimized Targeting via Smart Carriers

To improve the efficacy of targeted immunotherapy and reduce off-target adverse effects, drug or cell delivery systems precisely delivering therapeutic agents to the TME have been widely applied.^148^ The advantages of using drug delivery systems are obvious and manifold: delivery of multiple agents simultaneously, enabling synergistic effects,^149^ enhancing pharmacokinetics by improving the stability and half-life, and highly specific targeting achieved via functionalization with targeting ligands that recognize specific biomarkers on cancer cells or immune cells.^150^

**Figure 10:**
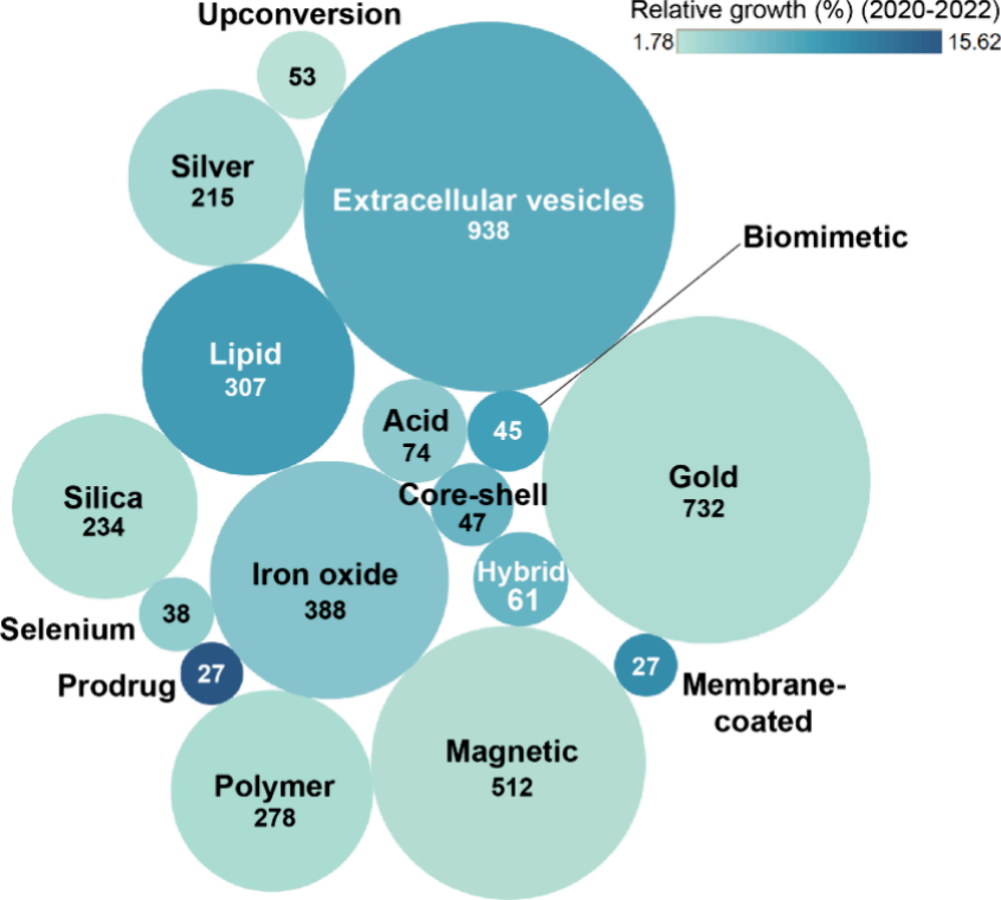
Distribution of drug delivery vehicles used in cancer immunotherapy. The size of the circles reflects the number of publications in the CAS Content Collection for the period 2000–2022 related to the delivery vehicle type. The color intensity reflects the relative publication growth rate for the past three years (2020–2022).

Frequently used delivery systems include nanoparticles, cell-based and exosome-based delivery systems, biomaterial-based implants, and injectable hydrogels and scaffolds. With attractive tunable size and surface properties, nanocarriers can increase the delivered drug’s overall therapeutic index that are either encapsulated or conjugated to the surfaces of nanoparticles. Materials such as lipids, metals, polymers, and others have been applied (Figure 10). Clinical research has demonstrated that a significant proportion of cancer patients exhibit insensitivity to immunotherapy, mostly because of the immunomodulatory exchanges between tumor cells and the immunosuppressive TME, managing the immune tolerance of tumors. Significant efforts have been concentrated on introducing nanosized carriers to cancer medicine.^151^ Targeted remodeling of the immunosuppressive TME using appropriately engineered nanoparticles provides a favorable strategy for enhancing the effectiveness of tumor immunotherapy^150,152^ and helps to overcome immune tolerance of tumors.^153^

### Nanoparticles (NPs)

Broadly speaking, nanodrug delivery systems can be divided into (1) passive targeting, transported to target sites through regular physiological processes with carriers including emulsions, liposomes, microcapsules, or microspheres; (2) active targeting carriers, the surfaces of which have been modified with specific ligands to enable binding to receptors on the target cells or organs; and (3) physical and/or chemical targeting carriers directed by external forces such as temperature, pH, or magnetic field.

Pharmaceutical nanocarriers can be prepared from lipids, polymers, metals, inorganics, and other materials. Nanomedicines target tumors via passive (also known as the Enhanced Permeation and Retention effect) and/or active mechanisms.^154^ Research and development in the field of cancer nanotherapeutics has experienced an exponential growth since the early 2000s, since the commercialization of the first-generation antitumor nanomedicines, including DOXIL (doxorubicin hydrochloride, Baxter)^155^ and Abraxane (paclitaxel, Bristol-Myers Squibb).^156^

1. **Lipid NPs** – Lipid NPs^157^ are currently the most widely used NPs and are exhibiting a higher growth rate as compared to other NPs in the field of cancer immunotherapy (Figure 2). Lipid-based NPs are superior to other nanosized drug delivery systems in minimizing systemic toxicity while maintaining adequate solubility^158^ and are thus the most common type of regulatory approved nanomedicines.^159^ They have been applied in drug delivery since the discovery of liposomes in the 1960s but marked several serious advancements: (i) with the introduction of PEGylation,^160^ which increased their circulation half-lives, further improving their effectiveness;^161^ (ii) with the discovery of the cationic/ionizable liposomes able to deliver anionic nucleic acids;^162^ as well as (iii) with the development of the solid lipid NPs and the nanostructured lipid carriers offering higher payload and stability and largely improved scalability.^163^ Thus, in cancer immunotherapy, lipid-based NPs could successfully deliver small molecules and mRNA therapies *in vivo* to achieve remarkable antitumor activity.
2. **Metal NPs** – Inorganic NPs provide an appropriate framework in which multiple modules can be combined to give multifunctional capabilities. Metallic NP formulations are particularly advantageous because of their potential for dense surface functionalization and capability for optical- or thermal-based therapeutic and diagnostic methods.^164^
3. **Magnetic iron oxide NPs** – Iron oxide NPs can generate heat when exposed to an alternating magnetic field, a property that has been utilized to induce cell death and stimulate an immune response in hyperthermia-based cancer treatment. Iron oxide NPs can also be used as contrast agents for magnetic resonance imaging, allowing for noninvasive tracking of immune cell migration and infiltration into tumor sites. In order to enhance their cellular uptake and effectiveness, these NPs can be modified with a specific coating, conjugated to drugs, proteins, enzymes, antibodies, or nucleotides, and can be directed to an organ, tissue, or tumor sites using external magnetic field. They can also be used in the development of dual-purpose probes for the *in vivo* transfection of siRNA.^165^
4. **Silver NPs** – Silver NPs, known for their antibacterial activity, are also known to enhance the antitumor effects of anticancer drugs in combination therapies, allowing use of lower doses to reduce cytotoxic effects and increase efficacy.^166^ They can thus operate as direct anticancer agents, as well as delivery platforms of various cytotoxic drugs, or enhance the anticancer performance of combinational partners upon chemo- or radiotherapy.^167^ Silver NPs can exhibit a plasmon resonance effect and generate heat when exposed to specific wavelengths.^168^ This property can be harnessed for photothermal therapy, where the localized heat generated by the NPs damages cancer cells and stimulates an immune response.
5. **Gold NPs** – Gold NPs are a multifunctional therapeutic modality, performing as targeted delivery systems for vaccines, nucleic acids, and immune antibodies, as theranostic agents, and as tools in photothermal cancer therapy. They have been successfully applied also in medical imaging, such as radiotherapy, magnetic resonance angiography, and photoacoustic imaging. Gold nanostructures, including nanoparticles, nanorods, nanocages, etc., are easily synthesizable in diverse shapes and sizes through various chemical, physical, or biological methods, which empowers their manageability, since even minor modifications of their size and shape can produce significant alterations in their functional properties, including biodistribution, metabolism, cytotoxicity, and immunogenicity.^169^ Similar to silver NPs, gold NPs can be utilized in photothermal therapy via localized surface plasmon resonance.^170^
6. **Silica NPs** – Mesoporous silica exhibits high porosity, appropriate biocompatibility, and facile surface functionalization. Silica NPs can be engineered to various shapes, sizes, and surface properties, making them versatile tools for targeted drug delivery, imaging, and immunomodulation.^171^ Since the introduction of submicrometer mesoporous silica, termed MCM-41,^172^ and its successful application as a nanocarrier,^173^ it has been regarded as a promising drug delivery system. Moreover, mesoporous silica exhibits a self-adjuvant property, significantly enhancing anticancer immunity without additional immunomodulators.^174^ Mesoporous silica has emerged as a prospective nanocarrier for cancer vaccines as well,^175^ alleviating antitumor immunity through dual loading of antigen and adjuvant on a single platform.^171^
7. **Polymeric NPs** – Polymeric nanocarriers are beneficial for immunotherapy approaches because they can be modulated with adequate physical properties, encapsulants, and surface ligands; they can be also tailored to co-deliver multiple therapeutic agents to cancer or immune cells.^176^ Various stimuli-responsive (e.g., enzyme-, pH- and redox-responsive) polymers, including natural and synthetic polymers, have been utilized as smart nanocarriers for immunotherapy applications. Redox-responsive polymeric nanohydrogels exhibiting tissue-like mechanical properties and high porosity have been extensively studied and shown to be effective in protecting payloads including protein drugs, gene therapeutics, and small-molecule anticancer drugs in blood circulation, as well as in their targeted release.^177^ The most commonly used natural polysaccharides include dextran, polysialic acid, hyaluronic acid, chitosan, and heparin, while polyvinylpyrrolidone, polyacrylamide, polyvinyl alcohol, polyethylene glycol (PEG), and PEG copolymers such as poloxamines, poloxamers, and polysorbates are preferred synthetic polymers.^176^ Smart polymeric NPs have been demonstrated to enhance tumor immunotherapy, to alleviate immunosuppression, and to prevent cancer cells from evading the immune system.^176,178^

### Exosomes

Superior innate stability, low immunogenicity, biocompatibility, and excellent capacity for membrane penetration allow exosomes to be valuable natural nanocarriers for efficient drug delivery.^179^ As important mediators of intercellular communications, exosomes are increasingly gaining interest in the context of cancer immunotherapy.^180^ Exosomes, either tumor-derived, comprising tumor-associated antigens, or derived from dendritic cells presenting antigens, can trigger immune activation, and therefore they can be used in developing anticancer vaccines.^181^ Moreover, tumor-derived exosomes hold information from primary cells and express complexes of MHC epitopes and co-stimulatory proteins; thus, they can activate CD8 T-cells, which offers unique therapeutic approaches for developing cancer vaccines.^182^ Exosomes participate in the formation of the cancer immunosuppressive microenvironment, so tumor exosome production control might be an effective treatment strategy. Exosomes also play a key role in PD-1/PD-L1 immune checkpoint inhibitor treatment.

### Others

A variety of other nanovehicle delivery systems for cancer immunotherapy are found in the CAS Content Collection: prodrug NPs,^183^ core–shell NPs,^184,185^ hybrid NPs,^186^ photon upconversion NPs,^187^ biomimetic NPs,^188^ cell membrane-coated NPs,^189^ deformable nanoconstructs,^190^ stimuli-responsive NPs,^191^ etc.

As compared to nanoparticle-mediated, noncovalent drug encapsulation, stimulus-responsive prodrug nanoparticles have a pharmaceutical advantage: they can be tuned to minimize drug leakage and to control drug release profiles through chemical linkers^192^ and have recently been developed as a strategy to enhance the effectiveness of cancer immunotherapy.^183^ Iron oxide–zinc oxide core–shell NPs have been successfully used to deliver carcinoembryonic antigen into dendritic cells while simultaneously acting as imaging agents.^184^ Gold–silver core–shell hybrid NPs have been reported to attenuate the tumor-cell-promoting activity of CAFs, leading to a prominent attenuation of metastatic dissemination *in vivo*.^185^ Hybrid NPs comprising two or more constituents with different compositions and properties, typically organic and inorganic, have been developed, combining the functions of participating materials and exhibiting immense potential in advancing cancer immunotherapy.^186^ Upconversion NPs devised for cancer therapy typically have employed photoinitiators and dyes to enhance reactive oxygen species by producing radicals in the tumor site through photodynamic therapy.^187^ Use of stimuli-responsive delivery systems can enable spatiotemporal control over drug release.

## Biomarkers: The Earlier Detected the Better

Early detection of cancer is critical for reducing its morbidity and mortality.^193^ Kinases, chemokines, transcription factors, and glycoproteins stand out as key players among the diverse classes of emerging biomarkers for various types of cancers due to their intricate involvement in cancer development, progression, and response to therapy.

In the realm of cancer immunotherapy, certain transcription factors have been recognized for their pivotal roles in orchestrating immune responses and influencing the TME. Research on transcription factors such as ENO1, SETD2, nectin-4, TROP2, gasdermin E (GSDME), TCF-7, and glutathione peroxidase 4 (GPX4) has gathered increased attention in the past three years, showing increased growth in journal and patent publications based on the CAS Content Collection (Figure 2). As understanding of the complex interplay between transcription factors and the immune system deepens, it can be expected that transcription factor-based biomarkers will play an increasingly important role in the quest for precision cancer immunotherapy.

ENO1, a glycolytic enzyme acting as a plasminogen receptor on cell surfaces, contributes to cancer cell proliferation, migration, invasion, and metastasis.^194^ ENO1 overexpression has been documented in a broad range of cancers including lung, breast, liver, and others and is usually associated with poor prognosis. This overexpression can contribute to the metabolic changes associated with cancer cells, as they regularly rely on glycolysis for energy production. In breast cancer, enhanced ENO1 expression has been associated with greater tumor size and a shorter disease-free interval.^195^ Patients with lung cancer overexpressing ENO1 also exhibited poor clinical outcomes, with shorter overall survival.^196^ ENO1 overexpression in hepatocellular carcinoma correlated positively with venous invasion.^197^ ENO1 overexpression in multiple cancer types, its localization at the cancer cell surface, and its targetability make this protein a rising cancer biomarker and potential target for therapeutic agents.^198^

SETD2 is a gene that encodes an enzyme involved in histone modification, specifically histone H3 lysine 36 (H3K36) methylation. This gene plays a crucial role in epigenetic regulation and has been implicated in various cellular processes, including DNA repair, transcriptional regulation, and chromatin organization. Mutations in SETD2 can be both loss-of-function and gain-of-function mutations, affecting its histone methylation activity. Frequent mutations have been reported in cancers like clear cell renal cell carcinoma,^199^ ALL,^200^ glioblastoma,^201^ and others. The correlation between SETD2 deleterious mutation and TMB has placed it in the spotlight as a promising prognostic biomarker for cancer ICI treatment.^56,202^

The cell adhesion protein nectin-4 also emerges as a favorable prognostic blood-based biomarker and is known to be overexpressed in several cancer types, including breast,^203^ ovarian,^204^ endometrial,^205^ urothelial,^206^ and certain subtypes of bladder cancer.^207^ High levels of nectin-4 expression have been correlated with more aggressive cancer behavior, poor prognosis, and shorter survival rates, making it also an emerging prognostic biomarker.^208^ For example, in a multivariate clinical analysis of triple-negative breast cancer survival rate, nectin-4 has been highlighted as a favorable breast cancer prognostic marker.^55^

Gasdermin E (GSDME), also known as DFNA5 (Deafness, Autosomal Dominant 5), is a protein that is involved in programmed cell death, specifically pyroptosis, a form of cell death in response to infection or cellular stress. GSDME has been found highly expressed in most malignant cancers, and an obvious relationship exists between GSDME levels and survival prognosis of cancer patients.^209^ Studies have also shown that GSDME methylation is a valuable molecular biomarker in cancer, specifically in breast cancer diagnoses,^210^ with a recent report suggesting its importance in a variety of other cancer types as well.^211^ The prominent expression and methylation properties of GSDME in diverse cancers make it an emerging favorable prognostic biomarker with a tremendous potential.

IGF2BP2, belonging to the family of RNA-binding proteins, plays a role in post-transcriptional regulation of gene expression by binding to specific target mRNAs, thereby influencing their stability and translation. While IGF2BP2 is more commonly associated with metabolic functions and type 2 diabetes, it has also been studied in the context of cancer.^212^ Its overexpression can lead to the stabilization and increased translation of specific mRNA targets and, in some cases, can promote tumorigenesis and cancer progression by enhancing the expression of oncogenic genes.^213^ In the context of hepatocellular carcinoma, IGF2BP2 overexpression may be linked to cancer development.^214^ The expression levels of IGF2BP2 in tumor tissues and its correlation with clinical outcomes are areas of active research.

Tumor mutational burden (TMB) has recently emerged as a significant and independent predictor of ICI treatment response to diverse tumor types. TMB is a measure of the number of mutations expressed by cancer cells and can arise due to various factors, such as exposure to environmental carcinogens or errors during DNA replication. Clinical studies have demonstrated that patients with high TMB tend to have better responses to ICIs and improved overall survival rates.^59^ These findings have led to the exploration of TMB as a biomarker to guide treatment decisions in immuno-oncology^215^ across various cancer types. By assessing a tumor’s mutational burden, clinicians can identify patients who are more likely to benefit from ICI therapy, thereby personalizing treatment approaches.^216^

Other biomarkers of apparent prognostic significance include TROP2, a transmembrane glycoprotein overexpressed in many cancers, including breast, lung, colorectal, pancreatic, and bladder cancers,^58^ and LC3B, a protein involved in the autophagy pathway. Autophagy is a highly regulated process that plays a critical role in maintaining cellular homeostasis. Higher levels of LC3B expression are associated with better patient outcomes, indicating an advantageous prognostic value.^217^

## Challenges in the Field

While it is undeniable that the continued development of immuno-oncology has been encouraging for cancer treatment, many challenges in the field persist. Some major challenges include (i) limited effectiveness in patient populations necessitating better methods to predict patient response, including identification of new biomarkers; (ii) development of resistance; (iii) lack of robust preclinical models; and (iv) immune-related adverse events (irEAs) as side effects.

The problem of limited efficacy has been the subject of much research, with several meta-analysis studies being published (for NSCLC^218^ and HNSCC^219^), and multiple theories have been proposed to account for it. The immune system is a highly complex and well-regulated system; as such, there are multiple components in place to act as fail-safes. In the context of ICIs, one proposed theory attributes reduced efficacy to other immune checkpoint molecules stepping up to play a compensatory role—using a combination approach is proposed to mitigate this phenomenon,^220^ though that is accompanied by its own set of challenges, and finding the most effective combinations and determining the optimal dosing schedules remain areas of ongoing research.^221^ Preclinical animal models have inherent limitations,^222^ and this can complicate discovery and testing of cancer immunotherapeutic drugs.

Resistance against immunotherapeutic strategies (intrinsic, extrinsic, or acquired^223^) can also limit effectiveness. A few proposed mechanisms for intrinsic resistance include decreased antigen expression, leading to escape from immunosurveillance, and heterogeneity among tumors, while mechanisms for acquired resistance include changes in the TME involving alterations in levels of TILs, CAFs, MDSCs, etc.^224^ A recent report suggests that the use of senolytic drugs capable of reducing levels of senescent cells^225^ before ICI treatment may help overcome resistance and boost effectiveness.^226^ A group of undesirable immune-related autoimmune events (irAEs) may occur upon administration of ICIs.^227^ irAEs can affect a wide variety of organ systems, can arise either during ICI treatment or even after completion of treatment, and can end up being chronic,^228^ all of which can detract from the use of ICIs. Treatment of irAEs involves administration of immunosuppressants, impacting the effectiveness of ICI therapy and sometimes accompanied by cessation of ICI therapy.^229^ Research efforts are directed toward bettering detection, management, and prevention of irAEs.^230^

For a more detailed take on challenges in cancer immunotherapy, please refer to Hegde and Chen^231^ and Taefehshokr et al.^232^ In addition to and perhaps tied to the above challenges, extremely high treatment cost is another crippling drawback of cancer immunotherapy, levying enormous economic burden on patients and putting this potentially lifesaving treatment option out of reach for many patients.^233^

## Conclusions

The field of immuno-oncology has shown continuous and accelerated growth since 2000, with clear signs of further expansion. The success of immunotherapeutic drugs in treatment of hard-to-treat cancers has given patients a new lease on life and speaks to the enormous potential of immuno-oncology. Accelerating interest means the volume of information (in terms of scientific publications) that is becoming available can be overwhelming. Gaining insights from large volumes of data is invaluable in guiding future growth of the field. Achieving this lofty goal though is extremely challenging.

We tackled this challenge using a novel NLP-based approach and created a “Trend Landscape Map”. The map is designed to be a comprehensive resource and provides information about emerging concepts in the field of immuno-oncology across various levels—right from a panoramic bird’s eye view down to zoomed-in views of specific concepts. The intrinsic value of the map has been enhanced by incorporating information about growth and size of the field, giving a nuanced view. The four major areas where most of the identified emerging concepts were clustered are types of targets, therapies, biomarkers, and cancers. Areas where relative growth is considerably higher than the absolute number of publications represent potential lacunae of interest. For example, while immune checkpoint molecules PD-1 and PD-L1 continue to show steady growth and are supported by the existence of several PD-1-targeted U.S. FDA-approved therapeutics, other immune checkpoint molecules such as TIGIT, B7-H3, A2AR, and LAG3 show high relative growths with low absolute numbers of publications. A few other key areas of emergence worth highlighting are CAR therapy, especially CAR-NK and CD19 CAR-T; cancer vaccines, especially RNA-based and nanovaccines; and lipid-based and membrane-coated nanoparticles as modes of delivery systems. Biomarkers and therapeutic targets in immuno-oncology are closely tied and show a large degree of overlap. A few examples of targetable biomarkers that we identified as emerging include ENO1, nectin-4, TROP2, PCSK9, LILRB1, CEACAM5, and the immune checkpoint molecule TIGIT.

To identify and understand the trends across various emerging concepts in cancer immunotherapy, we have leveraged substance data from the CAS Content Collection and CAS Registry consisting of >219 million substances. Our analysis of >3.2 million substances sheds light on interesting trends—namely that protein/peptide sequences appear to be of greater commercial importance than small molecules, as evidenced by the substantially higher proportion in patent publications. However, a more nuanced look at the protein/peptide sequence to small molecule ratio led to insights about substance classes currently being explored with respect to emerging targets. In addition, co-occurrences between selected emerging therapies and types of cancer display interesting overlaps, indicating the use of targets across various cancer types.

Given the wealth of information, systematic analysis allows for maximized learning from previously published data and discovery of hidden connections between various concepts and ideas. Gaining nuanced insights, establishing obscure connections, and identifying concepts in their early emergent phases are bound to aid in directing research efforts in specific directions. Furthermore, timely updates to trend landscape analyses can help track the emergence of identified concepts and allow for systematic updates of emerging ideas. We hope that the insights we gleaned from our NLP-based analysis will serve as a tool which can be utilized to guide further exploration of research in the immuno-oncology field.

## Supporting Information Available

Detailed description of Natural Language Processing-based methods utilized to identify emerging topics; Figure S1: Graphical representation of NLP-based analysis; Figure S2: Number of publications vs relative growth for identified emerging therapies in immuno-oncology; Figure S3: Number of publications vs relative growth for identified emerging biomarkers in immuno-oncology; Figure S4: Number of publications vs relative growth for individual immune checkpoint molecules in immuno-oncology; Figure S5: Estimated timeline of emergence of therapies in the field of immuno-oncology; Figure S6: Estimated timeline of emergence of biomarkers in the field of immuno-oncology; Figure S7: Number of journal and patent publications related to immune checkpoint molecules over the past two decades (2001–2022); Figure S8: Sankey plot of co-occurrences of selected emerging types of therapies (left) and types of cancer (liquid tumors; center) and immune checkpoint molecules (right) in journal publications; Figure S9: Number of substances associated with journal and patent publications in the field of immuno-oncology over the a decade (2013–2022); Figure S10: Protein/peptide sequences to small molecule substance ratios for emerging therapeutic targets associated with publications; Figure S11: Commercial interest in immuno-oncology (PDF)

## Abbreviations Used

A2AR: adenosine A2A receptor
ACT: adoptive cell therapy
ADC: antibody–drug conjugate
ALL: acute lymphoblastic leukemia
AML: acute myeloid leukemia
ARG1: arginase 1
BCG: Bacillus Calmette-Guerin
BCL2: B-cell lymphoma 2
BCMA: B-cell maturation antigen
BTC: biliary tract cancer
BTLA: B and T lymphocyte attenuator
CAF: cancer-associated fibroblast
CAR-M: chimeric antigen receptor macrophage
CAR-NK: chimeric antigen receptor NK cell
CAR-T: chimeric antigen receptor T cell
CC: cervical cancer
CDC20: cell division cycle protein 20
CDK: cyclin-dependent kinase
CEACAM5: carcinoembryonic antigen cell adhesion molecule 5
CHE: Switzerland
cHL: classical Hodgkin’s lymphoma
CHN: China
COX-2: cyclooxygenase-2
CRC: colorectal cancer
cSCC: cutaneous squamous cell carcinoma
CSF-1R: colony-stimulating factor 1 receptor
CTLA-4: cytotoxic T-lymphocyte-associated antigen 4
DFNA5: Deafness, Autosomal Dominant 5
DEU: Germany
DLBCL: diffuse large B-cell lymphoma
dMMR: deficient mismatch repair
EC: esophageal cancer
ENO1: α-enolase 1
FAP: fibroblast activation protein
FDA: U.S. Food and Drug Administration
FLT3: fms-like tyrosine kinase 3
FRA: France
GBR: United Kingdom
GPC3: glypican-3
GSDME: gasdermin E
H3K36: histone H3 lysine 36
HBV: hepatitis B virus
HCC: hepatocellular carcinoma
HDAC: histone deacetylase
HER2: human epidermal growth factor receptor 2
HNSCC: head and neck squamous cell cancer
HPK: hematopoietic progenitor kinase
HRPC: hormone-refractory prostate cancer
ICI: immune checkpoint inhibitor
ICOS: inducible costimulators
IDO: indoleamine 2,3-dioxygenase
IFN-γ: interferon gamma
ILC: innate lymphoid cell
JAK: janus kinase
JPN: Japan
KOR: South Korea
LAG3: lymphocyte-activation gene 3
LILRB1: leucocyte immunoglobulin-like receptor B1
MCC: merkel cell carcinoma
MCL: mantle cell lymphoma
MHC: major histocompatibility complex
MM: multiple myeloma
MPM: malignant pleural mesothelioma
mTOR: mammalian target of rapamycin
NEK2: NIMA-related kinase 2
NHL: non-Hodgkin’s lymphoma
NK: natural killer
NLP: natural language processing
NPM1: nucleophosmin 1
NP: nanoparticle
NSCLC: non-small-cell lung cancer
PARP: poly-ADP-ribose polymerase
PCSK9: proprotein convertase subtilisin/kexin type 9
PD-1: programmed cell death 1 receptor
PD-L1: programmed cell death ligand 1
PEG: polyethylene glycol
PI3K: phosphoinositide 3-kinase
PKB: protein kinase B
PKM2: pyruvate kinase M2
PMBCL: primary mediastinal large B-cell lymphoma
RCC: renal cell carcinoma
sc-Fv: single-chain variable fragment
SCLC: small-cell lung cancer
SETD2: SET domain containing 2
TAA: tumor-associated antigen
TAM: tumor-associated macrophage
TBK1: TANK-binding kinase 1
TDO: tryptophan 2,3-dioxygenase
TIGIT: T cell immunoreceptor with Ig and ITIM domains
TIL: tumor infiltrating lymphocyte
TLR: toll-like receptor
TM: transmembrane
TMB: tumor mutation burden
TMB-H: tumor mutational burden-high
TME: tumor microenvironment
TNBC: triple negative breast cancer
T_reg_: regulatory T
TRK: receptor tyrosine kinase
TROP2: trophoblast cell-surface antigen 2
TSA: tumor surface antigen
UCEC: uterine corpus endometrial carcinoma
USA: Unites States
VISTA: V-domain immunoglobulin suppressor of T cell activation
WHO: World Health Organization
