# Supplementary material for: Emerging Targets and Therapeutics in Immuno-Oncology: Insights from Landscape Analysis
Source: J Med Chem. 2024 May 24;67(11):8519–44. doi: 10.1021/acs.jmedchem.4c00568 (PMC11181335; doi:10.1021/acs.jmedchem.4c00568)
Supplement: Supplementary file 1 — jm4c00568_si_001.pdf [file jm4c00568_si_001.pdf]

Supporting Information for

Emerging Targets and Therapeutics in Immuno-Oncology: Insights from Landscape Analysis

Kavita A. Iyer,[1] Julian Ivanov,[2] Rumiana Tenchov,[2] Krittika Ralhan,[1] Yacidzohara Rodriguez,[2] Janet Sasso,[2] Sabina Scott,[2] and Qiongqiong Angela Zhou[2]*

[1]ACS International India Pvt. Ltd., Pune 411044, India

[2]CAS, A Division of the American Chemical Society, Columbus, Ohio 43210, United States

*Corresponding author, email: qzhou@cas.org

Contents of SI:

- Methods
- Figures S1 to S11

Methods

- **Data extraction**

A comprehensive search query was crafted by subject matter experts to encompass the field of immuno-oncology and included the following terms: cancer immunotherapy, immune-oncology, anti-tumor immune response, anti-tumor immunity, tumor antigen presentation, cancer immunosurveillance, tumor-infiltrating immune cell, tumor cell recognition, pathogen-associated molecular pattern, damage-associate molecular patterns, immune signaling, carcinoma, immuno?, antibody cancer immunotherapy, immuno-oncology, cancer immunoediting, antigen presenting cell cancer, granzyme B cancer, MHC cancer, apoptosis resistance cancer immunotherapy, antibody drug conjugate, antibody-drug conjugate cancer, chimeric antigen receptor, adoptive immunotherapy cancer, cell therapy, T cell receptor, TCR, NK cell and therapy cancer, drug development and immunotherapy cancer.

Further refinement was achieved by combining above terms with the following terms: immunology, immune cell, antibody, immune response, immunotherapy, immune checkpoint, oncology, neoplasm, cancer.

The search query resulted in over 350,000 documents, which were extracted from CAPLUS database. Retrieved dataset included journal articles, patents, conference proceedings, dissertations, and preprints published from January 2000 through December 2022. From the CAS Content Collection (CAS, n.d.), the following information was extracted: title–abstract and claims (for patents), CAS indexing and concept approaches (based on full-text content), the year of publication, type of document, CAS section and subsection, up to date citations, name of organizations and countries in journal publications and patent assignees and assignee countries for patent publications, which includes patents published by 97 patent offices around the world. In addition, substance data was retrieved and included information about role indicators, such as THU for therapeutic use, PAC for pharmacological activity and PKT for pharmacokinetics, as well as information about substance class such as small molecule, protein/peptide sequences etc. to analyze trends of substances with therapeutic potential. Substance data was restricted to the last decade (2012-2022).

- **Natural Language Data processing**

For the detailed methodology of NLP analysis please refer to Ivanov *et al.*[1] A brief description is provided below:

We identified candidate phrases from the abstracts and titles (and claims for patent publications) that were anywhere between 1 to 6 words in length following removal of common English stop words. A crucial criterion for identification of phrases was appearance in at least 20 documents. This resulted in reduction of 119 million candidate phrases down to 338,054. These phrases were then subject to multiple rounds of manual perusal by subject matter experts to: 1. eliminate obvious noise, 2. group similar phrases and 3. classify phrases in to a hierarchical system. The metric we relied on to sift through and identify emerging concepts were the average publication and citation rates. The average publication rate is the difference between the number of publications each year and the previous year. The relative growth rate is the normalized average publication rate (with respect to number of documents) and was calculated as follows:

$$\text{dPub/dt 2020} = (\text{No. of publications in 2020} - \text{No. of publications in 2019})$$
$$\text{dPub/dt 2021} = (\text{No. of publications in 2021} - \text{No. of publications in 2020})$$
$$\text{dPub/dt 2022} = (\text{No. of publications in 2022} - \text{No. of publications in 2021})$$

$$\underset{(2020\text{-}2022)}{\text{Relative growth}} = \frac{\text{dPub/dt 2020} + \text{dpub/dt 2021} + \text{dPub/dt 2022}}{\text{Total number of publications (2020-2022)}}$$

We also estimated the year of emergence for each of the identified concepts by defining it as the point of time at which the average publication rate was at 10% of its maximal value. In addition, we calculated the average fold increase in publications over 2020-2022 as follows:

$$\text{Fold increase in 2020} = (\text{No. of publications in 2020} / \text{No. of publications in 2019})$$
$$\text{Fold increase in 2021} = (\text{No. of publications in 2021} / \text{No. of publications in 2020})$$
$$\text{Fold increase in 2022} = (\text{No. of publications in 2022} / \text{No. of publications in 2021})$$

$$\underset{(2020\text{-}2022)}{\text{Average fold increase}} = \frac{\text{Fold increase in 2020} + \text{Fold increase in 2021} + \text{Fold increase in 2022}}{3}$$

One caveat of our analysis was the inability to determine the context of emergence in the early stages requiring substantial manual intervention to validate. An area of potential improvement would be to expand the repertoire of common words beyond English stop words to include common scientific words – this would greatly reduce initial noise levels leaving behind only pertinent terms. Finally, development of automated initial screening of data would considerably diminish the time required to identify emerging concepts.

For the growth plots over time and co-occurrence analysis, data was gathered by searching for terms in the title, abstract, claims (for patent publications) and CAS indexed terms. Search terms included permutation combinations of multiple keywords including abbreviations to ensure complete coverage while ensuring low noise levels.

The Trend Landscape Map was designed and created using Abode Illustrator. Other data analysis about journal and patent publications and substance data was performed using primarily Tableau. Figures were prepared using a combination of Tableau, Microsoft Excel and Adobe Illustrator.

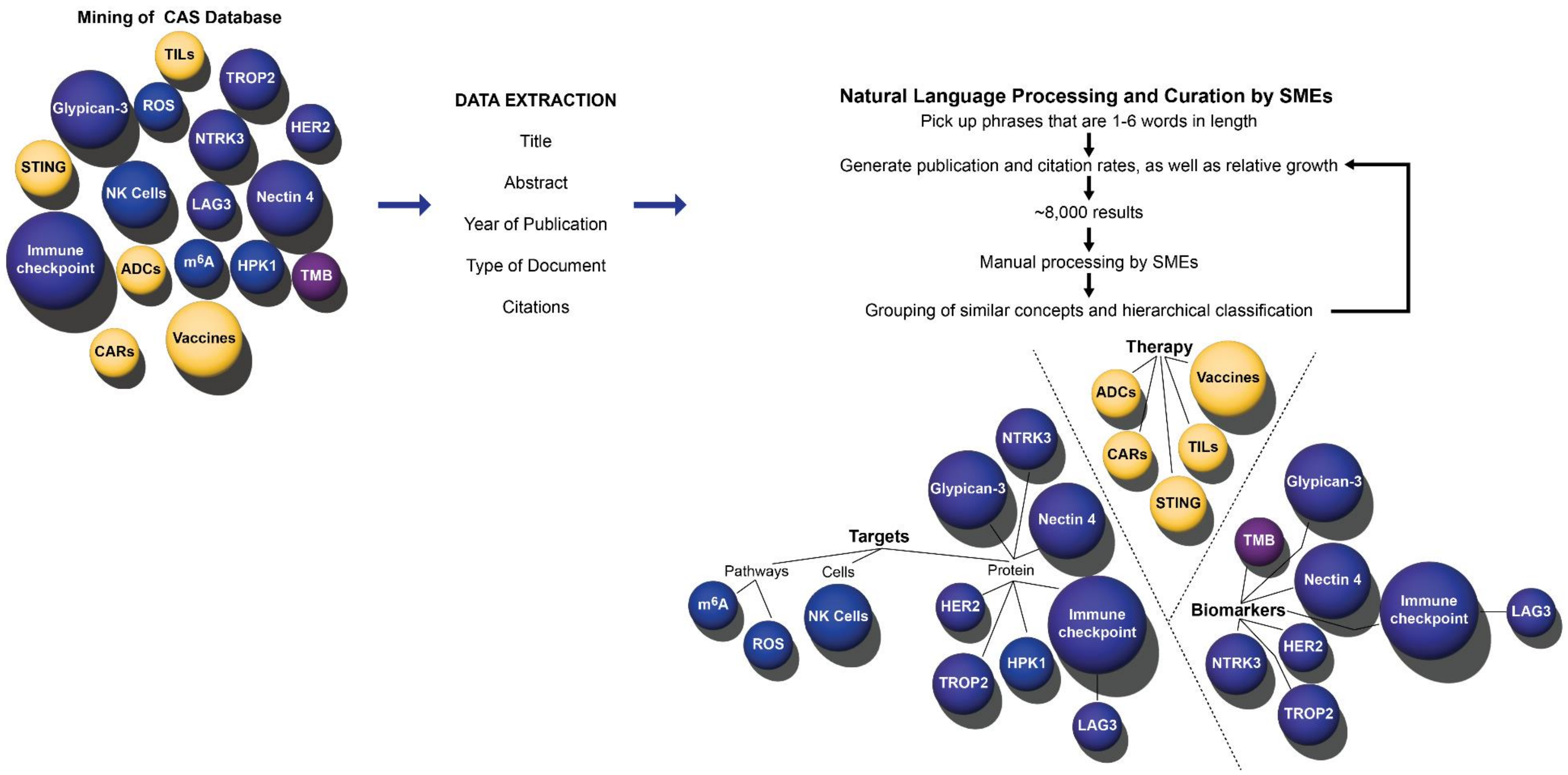

Figure S1. Graphical representation of workflow used to identify emerging concepts in immuno-oncology from data extracted from the CAS Content Collection. The analysis incorporated Natural Language processing (NLP)-based methods along with extensive manual curation by subject matter experts.

A

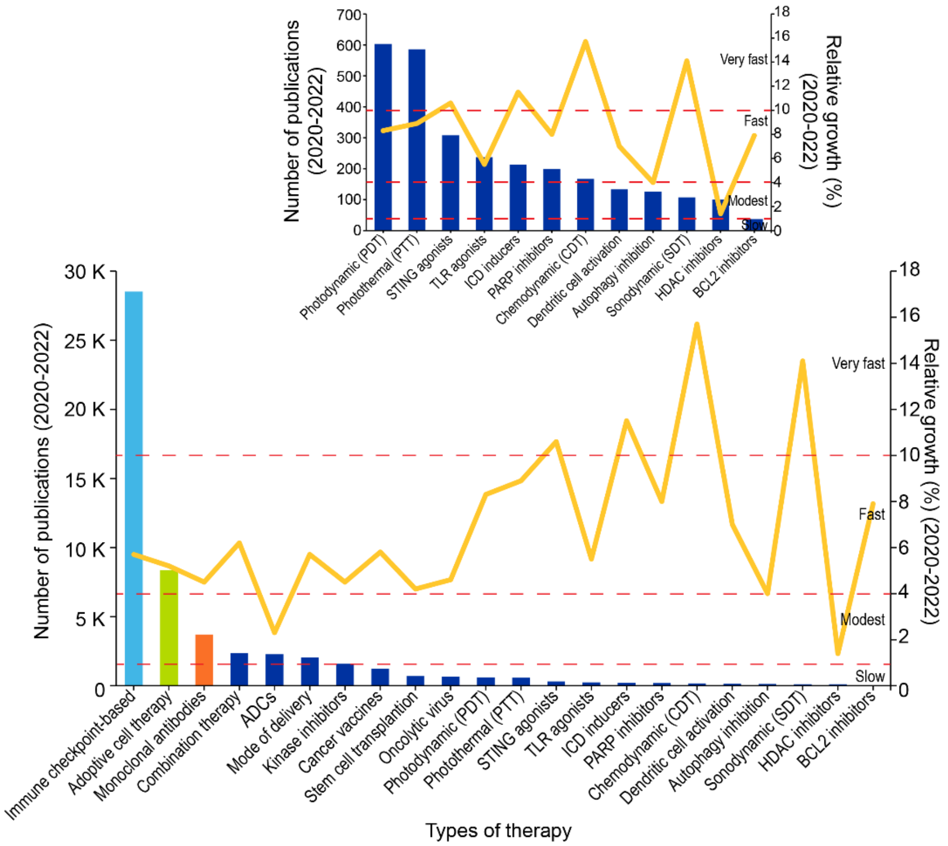

B

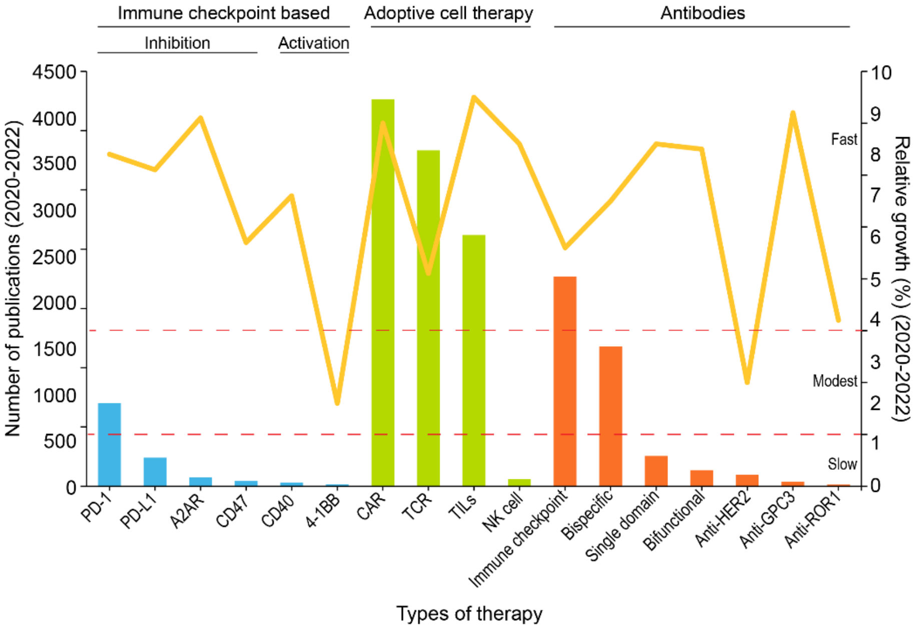

Figure S2. Comparison between number of publications (colored bars) and relative growth rate (yellow line) for (A) identified emerging therapies in immuno-oncology and (B) for individual members of selected therapy groups over 2020-2022 for data retrieved from CAS Content Collection. Blue-, green- and orange-colored bars representing immune-checkpoint based, adoptive cell therapy and monoclonal antibodies, respectively, in panel A are further broken down into individual members in panel B. Therapies can be divided into 4 categories of growth – very fast (>10%), fast (4-10%), modest (1-4%) and slow (<1%).

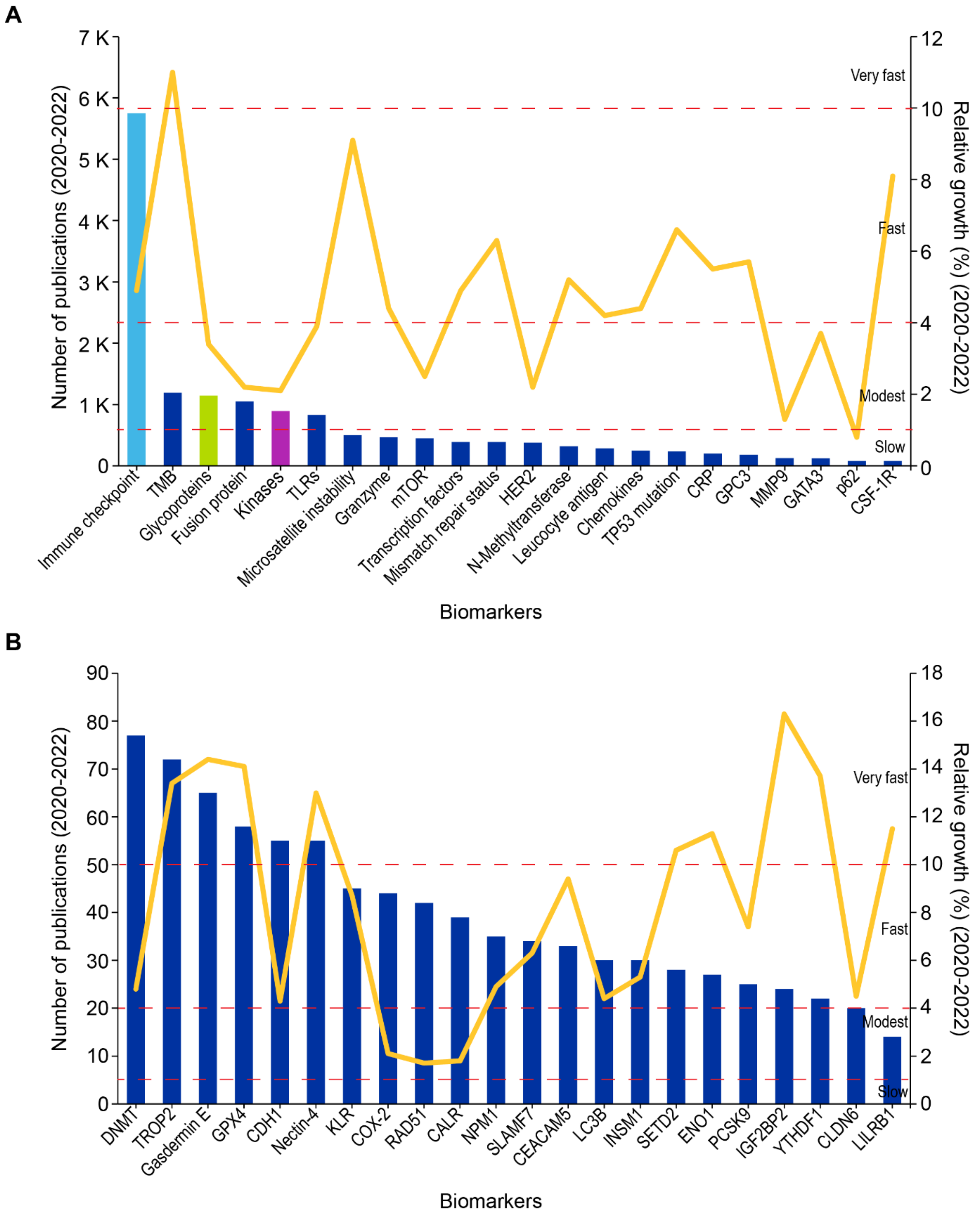

Figure S3. (A) and (B) Comparison between number of publications (colored bars) and relative growth rate (yellow line) over 2020-2022 for identified emerging biomarkers in immuno-oncology for data retrieved from CAS Content Collection. Biomarkers can be divided into 4 categories of growth – very fast (>10%), fast (4-10%), modest (1-4%) and slow (<1%). Blue-, green-, and magenta-colored bars in panel A representing immune checkpoint molecules, glycoproteins, and kinases, respectively, are further broken down into individual members in Supplementary Figure 4

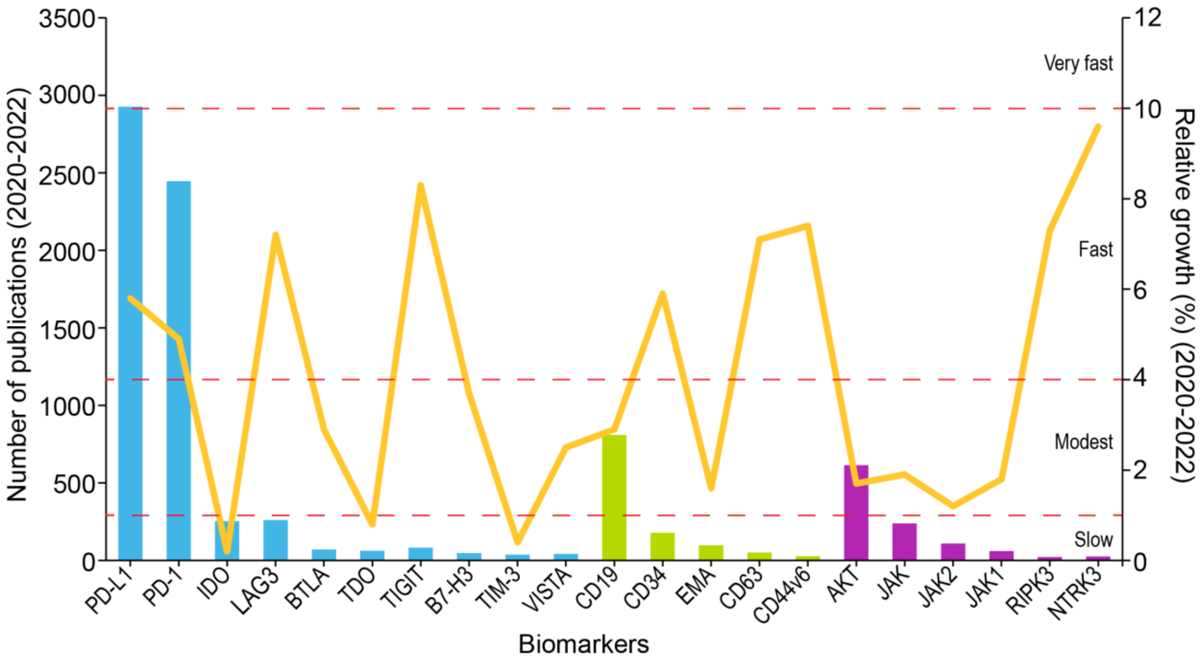

Figure S4. Comparison between number of publications (colored bars) and relative growth rate (yellow line) for individual immune checkpoint molecules (blue-colored bars), glycoproteins (green-colored bars) and kinases (magenta-colored bars) identified as emerging biomarkers in immuno-oncology for the period 2020-2022 for data retrieved from CAS Content Collection. Biomarkers can be divided into 4 categories of growth – very fast (>10%), fast (4-10%), modest (1-4%) and slow (<1%).

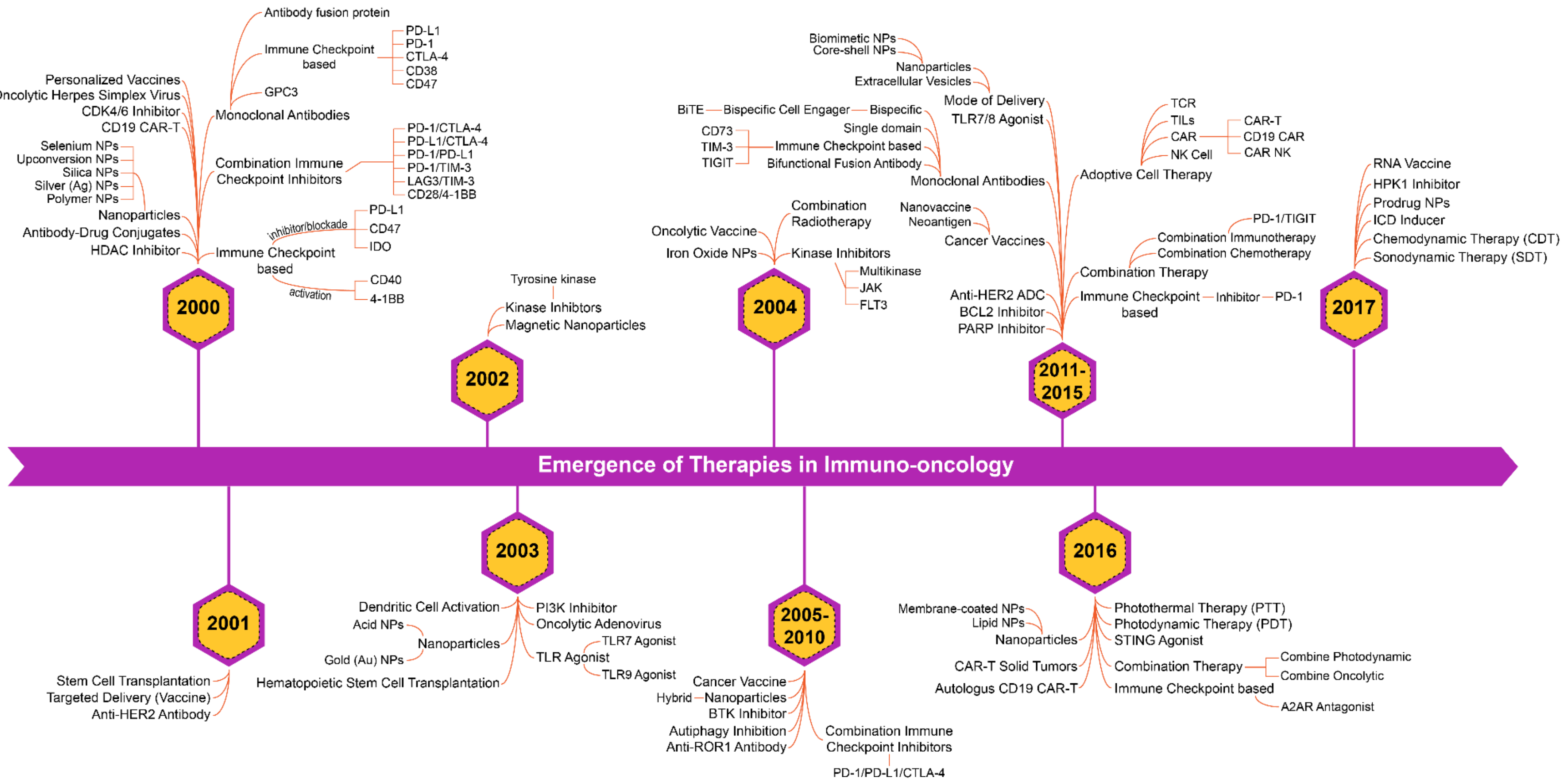

Figure S5. Estimated timeline of emergence of therapies in the field of immuno-oncology based on NLP analysis of >350K publications from the CAS Content Collection for the period 2000-2022.

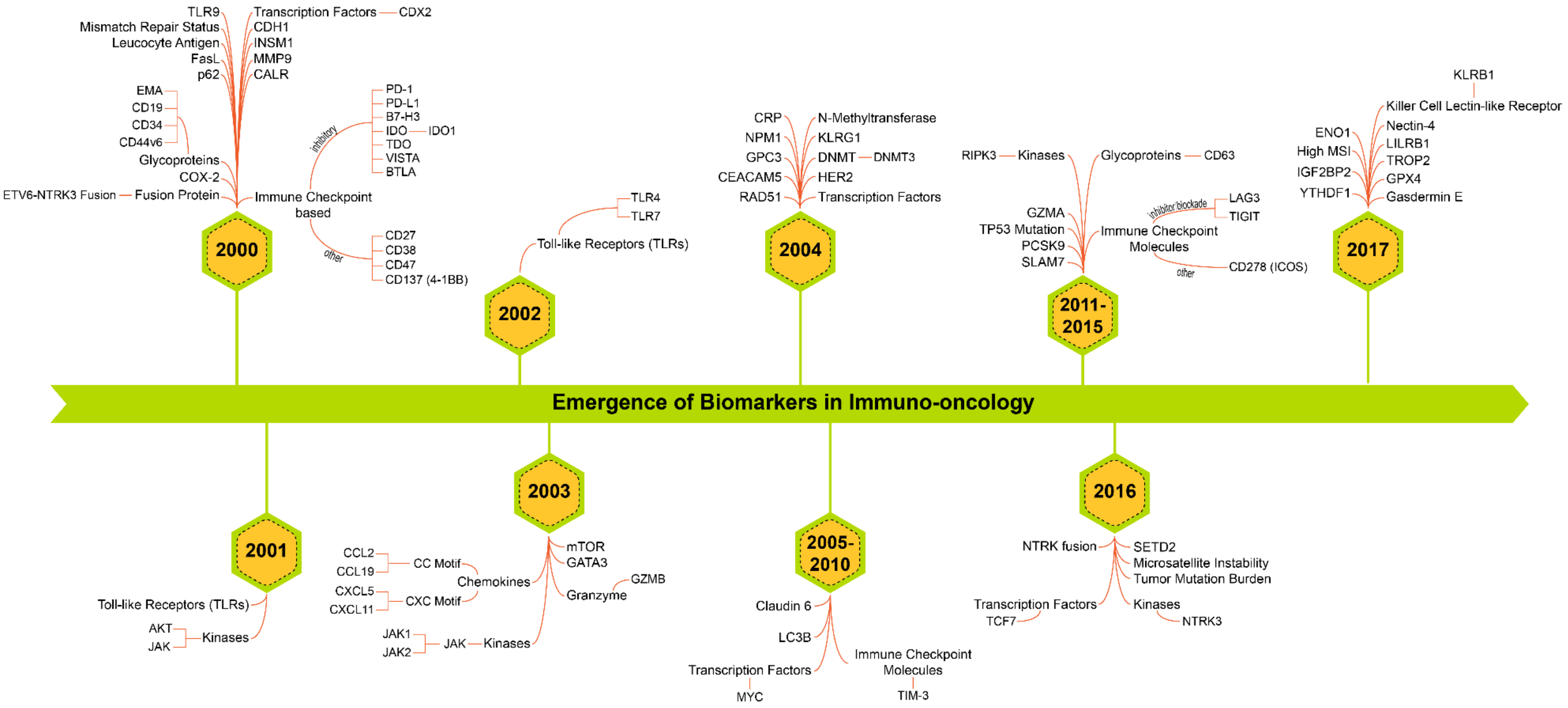

Figure S6. Estimated timeline of emergence of biomarkers in the field of immuno-oncology based on NLP analysis of >350K publications from the CAS Content Collection for the period 2000-2022.

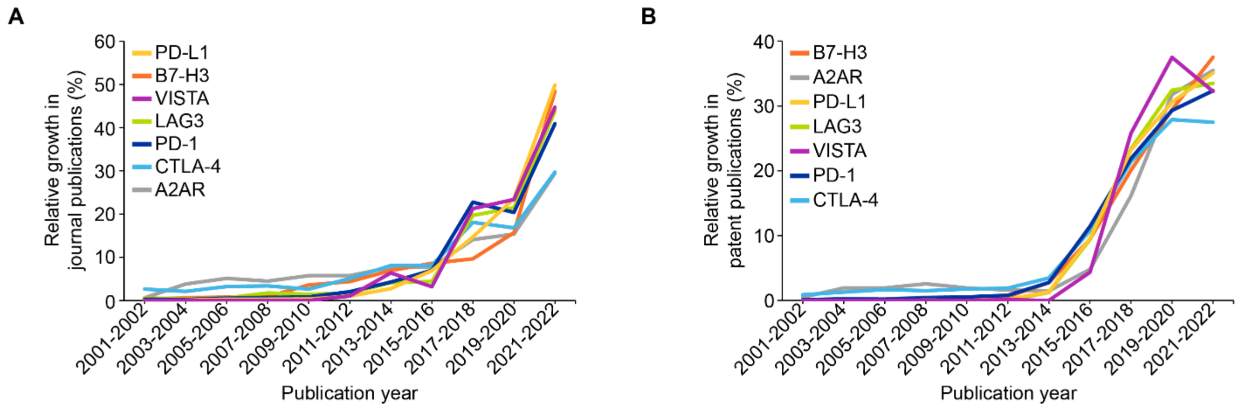

Figure S7. Growth of immune checkpoint molecules over the last two decades in (A) journal and (B) patent publications. Data includes publications from the CAS Content Collection for the period 2001-2022.

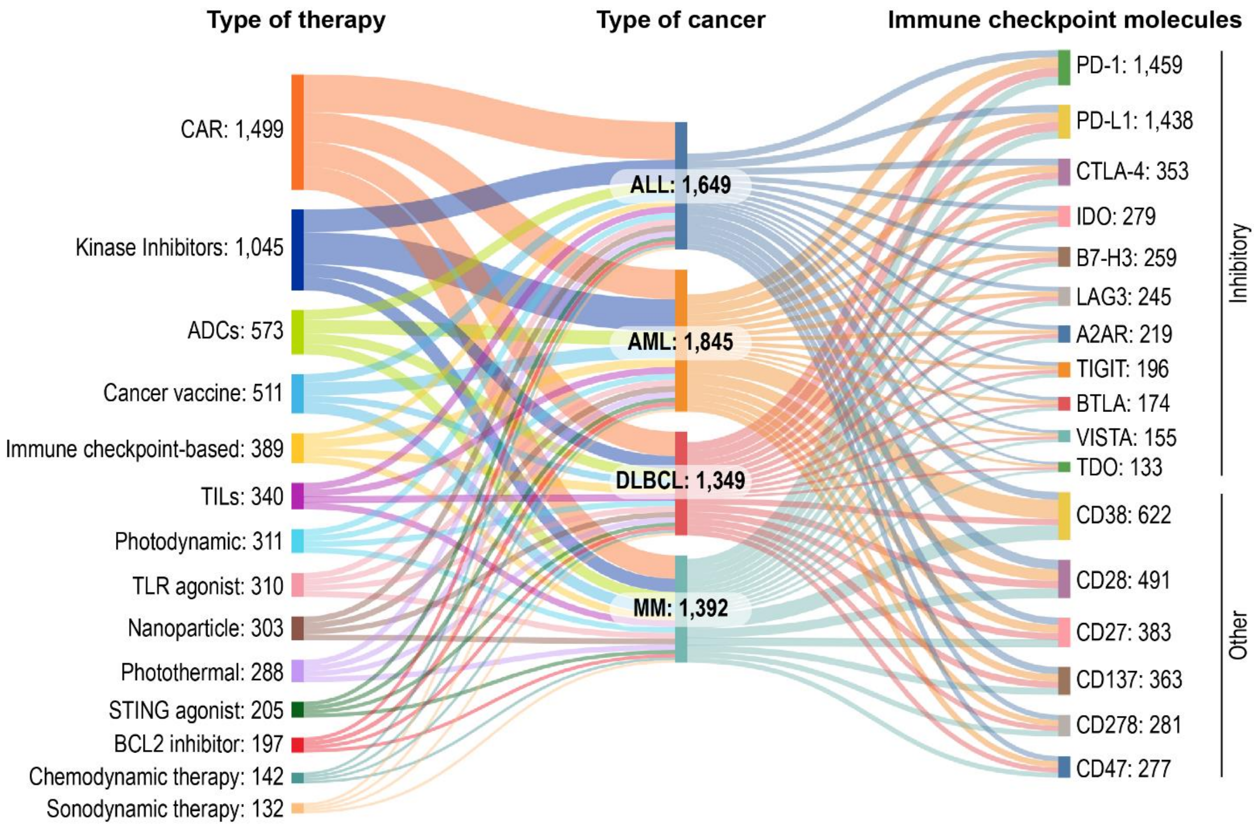

Figure S8. Co-occurrences of selected emerging types of therapies (left) and types of cancer (liquid tumors center) and immune checkpoint molecules (right) in journal publications depicted using a Sankey plot. Data includes journal publications from the CAS Content Collection for the period 2000-2022. Abbreviations: CAR – **C**himeric **A**ntigen **R**eceptor, ADCs – **A**ntibody-**D**rug **C**onjugates, TILs – **T**umor **I**nfiltrating **L**ymphocytes, TLR – **T**oll-**l**ike **R**eceptor, BCL2 – **B**-**c**ell **L**ymphoma **2**, ALL – **A**cute **L**ymphoblastic **L**eukemia, AML – **A**cute **M**yeloid **L**eukemia, DLBCL – **D**iffuse **L**arge **B**-**C**ell **L**ymphoma, MM – **M**ultiple **M**yeloma

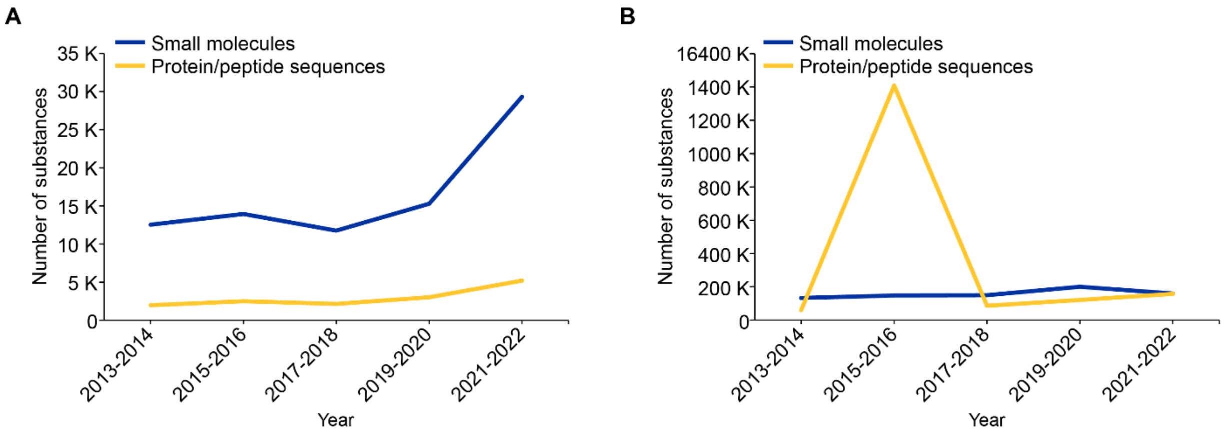

Figure S9. Growth in number of substances – small molecules and protein/peptide sequences – associated with (A) journal and (B) patent publications. Data is limited to substances with a therapeutic role for the period 2013-2022 in the field of immuno-oncology from the CAS Registry and CAS Content Collection.

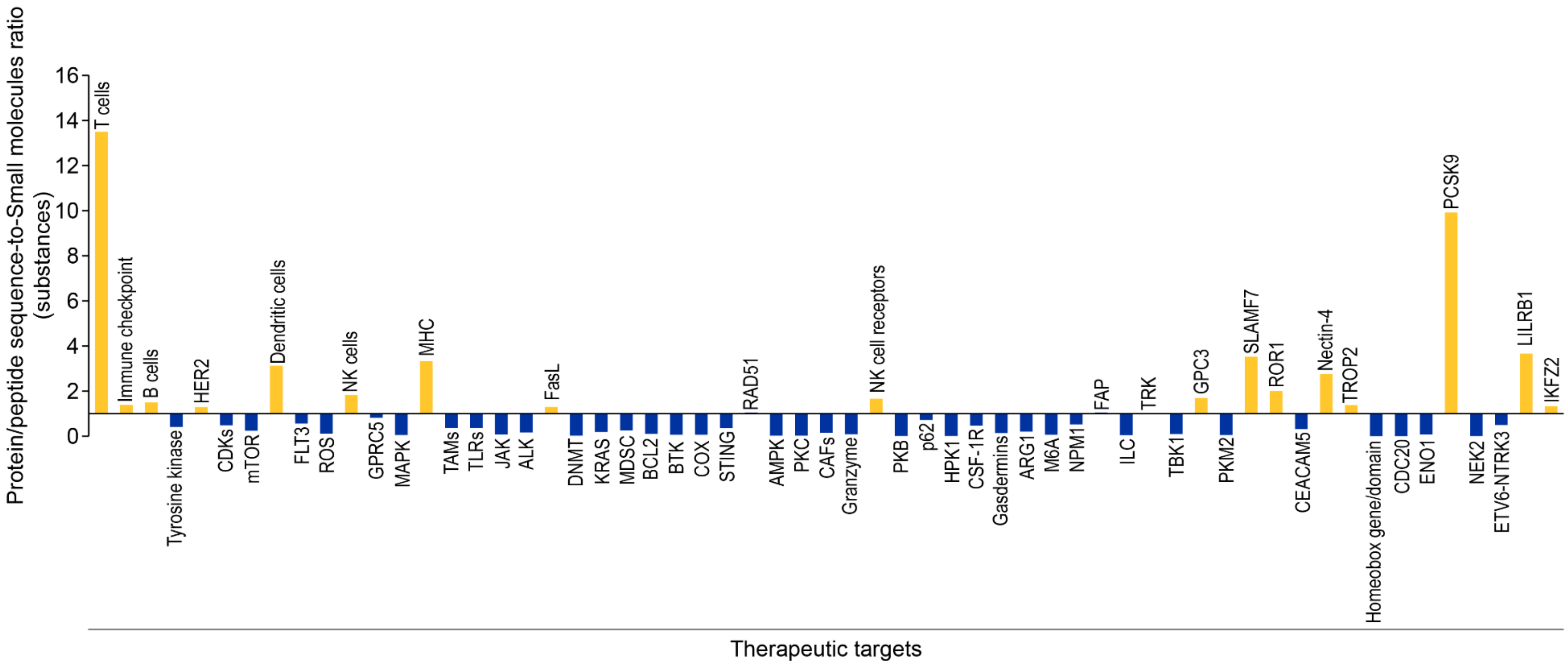

Figure S10. Protein/peptide sequences to small molecule substance ratios for emerging therapeutic targets associated with publications. Data is limited to substances with a therapeutic role for the period 2012-2022 in the field of immuno-oncology from the CAS Registry and CAS Content Collection.

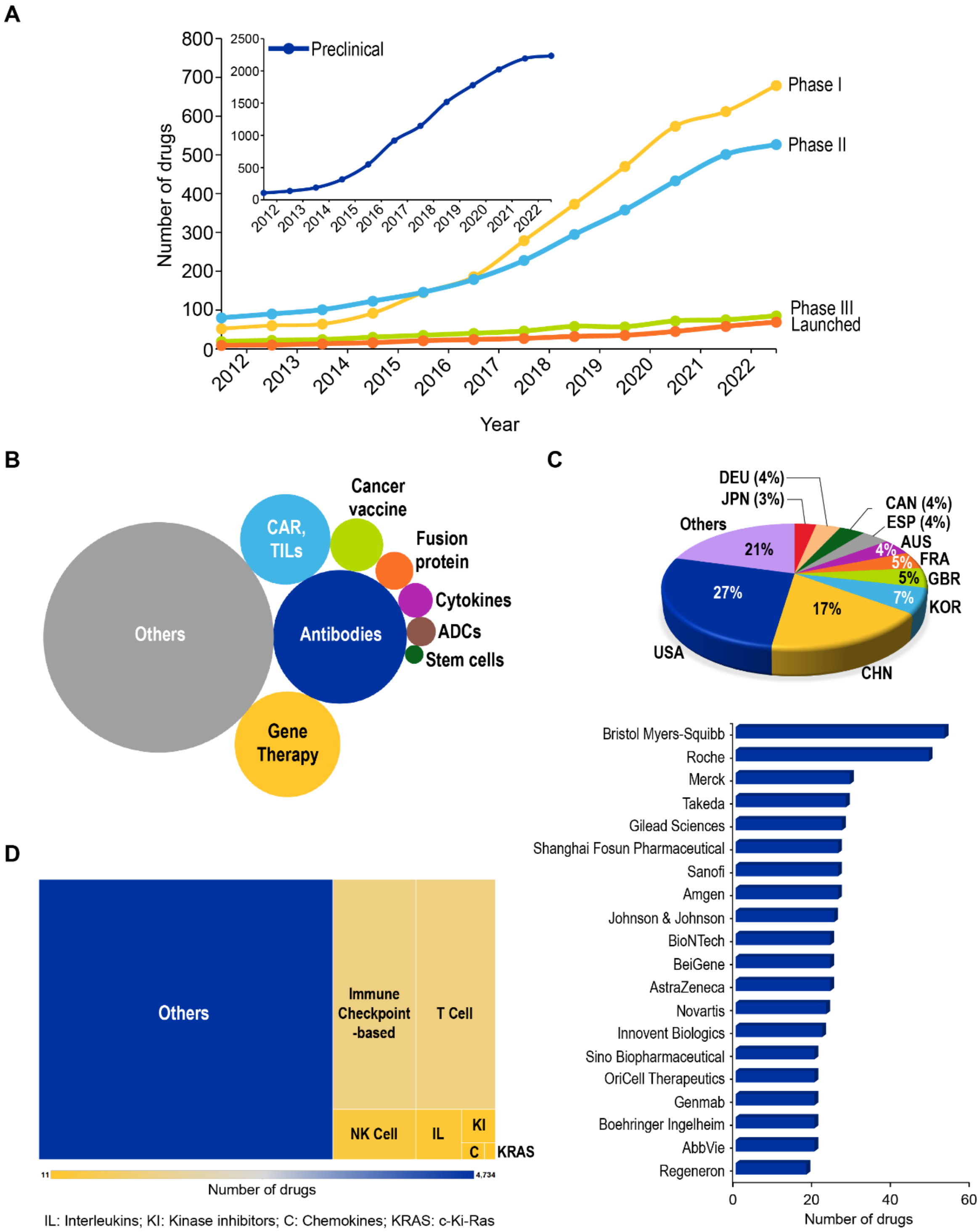

Figure S11. Overview of drugs in the pipeline in the field of immuno-oncology (data retrieved from Pharmaprojects). (A) Growth in number of drugs in the development pipeline (preclinical, phases I, II and III) as well as drugs that have been launched in the market. (B) Bubble chart showing number of drugs across various therapeutic classes in immuno-oncology. Size of the circle corresponds to number of drugs. (C) Geographical distribution of companies actively developing immuno-oncological therapeutics. Bar graph shows the top 20 companies in terms of number of drugs currently in the pipeline. (D) Heat map showing number of drugs across various mechanisms of action in immuno-oncology. Size and color correspond to number of drugs.

References

(1) Ivanov, J.; Lipkus, A.; Chen, H.; Aultman, C.; Iyer, K. A.; Tenchov, R.; Zhou, Q. Emerging topics – bibliometric-based methodology. 2023.
